# Structural insights into spliceosome fidelity: DHX35–GPATCH1- mediated rejection of aberrant splicing substrates

**DOI:** 10.1038/s41422-025-01084-w

**Published:** 2025-02-28

**Authors:** Yi Li, Paulina Fischer, Mengjiao Wang, Qianxing Zhou, Aixia Song, Rui Yuan, Wanyu Meng, Fei Xavier Chen, Reinhard Lührmann, Benjamin Lau, Ed Hurt, Jingdong Cheng

**Affiliations:** 1 Minhang Hospital & Institutes of Biomedical Sciences, Shanghai Key Laboratory of Medical Epigenetics, International Co-laboratory of Medical Epigenetics and Metabolism, Fudan University, Shanghai, China; 2 Heidelberg University Biochemistry Center (BZH), Heidelberg, Germany; 3 Cellular Biochemistry, Max-Planck-Institute for Multidisciplinary Sciences, Göttingen, Germany; 4 Molecular Systems Biology Unit, European Molecular Biology Laboratory (EMBL), Heidelberg, Germany

**Keywords:** Transcription, Cryoelectron microscopy

## Abstract

The spliceosome, a highly dynamic macromolecular assembly, catalyzes the precise removal of introns from pre-mRNAs. Recent studies have provided comprehensive structural insights into the step-wise assembly, catalytic splicing and final disassembly of the spliceosome. However, the molecular details of how the spliceosome recognizes and rejects suboptimal splicing substrates remained unclear. Here, we show cryo-electron microscopy structures of spliceosomal quality control complexes from a thermophilic eukaryote, *Chaetomium thermophilum*. The spliceosomes, henceforth termed B*^Q^, are stalled at a catalytically activated state but prior to the first splicing reaction due to an aberrant 5’ splice site conformation. This state is recognized by G-patch protein GPATCH1, which is docked onto PRP8-EN and -RH domains and has recruited the cognate DHX35 helicase to its U2 snRNA substrate. In B*^Q^, DHX35 has dissociated the U2/branch site helix, while the disassembly helicase DHX15 is docked close to its U6 RNA 3’-end substrate. Our work thus provides mechanistic insights into the concerted action of two spliceosomal helicases in maintaining splicing fidelity by priming spliceosomes that are bound to aberrant splice substrates for disassembly.

## Introduction

The spliceosome, a multi-megadalton ribonucleoprotein (RNP) complex, catalyzes nuclear pre-mRNA splicing, where introns are removed and exons are ligated together.^1–4^ Highly dynamic RNA–RNA and RNP networks form the active site of the spliceosome through a series of compositional and conformational rearrangements driven by RNA helicases,^5–7^ providing the necessary flexibility and accuracy of the splicing process.

Introns are recognized by the 5′ splice site (5′ss), branch site (BS) and 3′ splice site (3′ss),^8^ and are excised in two sequential trans-esterification reactions. Each intron removal requires an orchestrated construction of the pre-catalytic spliceosome (B complex) by assembling the small nuclear RNPs (snRNPs: U1, U2, and the U4/U6.U5 tri-snRNP) and numerous non-snRNP proteins. Complex B undergoes extensive compositional and conformational rearrangements, including dissociation of U4 snRNA, yielding the activated B^act^ complex. The latter is converted into a catalytically active spliceosome (designated B*) that catalyzes step I of splicing, yielding the cleaved 5′ exon and intron-3′ exon lariat intermediates. At this stage, the spliceosomal C complex is generated and after additional rearrangements, the C* complex catalyzes step II splicing, resulting in ligation of the 5’ and 3’ exons and excision of the intron lariat, and transition to the post-catalytic (P) state. Finally, the mRNA is released and the terminal spliceosome state, the intron-lariat spliceosome (ILS), is produced and subsequently disassembled. Cryo-electron microscopy (cryo-EM) studies of the different spliceosomal states, such as from *Saccharomyces cerevisiae* (referred to as baker’s yeast or *sc* in this study) and human cells, have provided the structural basis for the splicing mechanisms and offered new insights into the complex dynamics governing the transitions between different spliceosome states.^1,9–11^

To ensure fidelity, the spliceosome needs to recognize and discard suboptimal pre-mRNA substrates while it assembles, is activated and eventually catalyzes the pre-mRNA splicing. DEAD- and DEAH-box RNA helicases have been implicated in quality control mechanisms during the splicing cycle.^12^ They guarantee that only correctly spliced mRNAs are released from the spliceosome for nuclear export by promoting conformational changes that lead to the transition of the catalytic to the post-catalytic spliceosome while simultaneously antagonizing suboptimal substrates.^13^ Exon–intron boundaries are largely defined during early assembly steps, which is crucial for the fidelity of the splicing reaction. The 5’ss, BS and 3’ss are initially recognized in an ATP-independent manner by the U1 snRNP and splicing factors within the so-called E complex.^14^ Formation of complex A, which involves recruitment of U2 snRNP and the U2/BS base pair interaction, requires the RNA helicases UAP56 (*sc*Sub2) and DDX46 (*sc*Prp5), which, among others, also proofreads proper BS site recognition.^15,16^ The RNA helicases DHX16 (*sc*Prp2), DHX38 (*sc*Prp16) and DHX8 (*sc*Prp22) are involved in the branch formation (B^act^ to B*), exon joining (C to C*) and mRNA release (P to ILS), respectively.^7,12^ Moreover, DHX15 (*sc*Prp43) plays a crucial role in the disassembly of the terminal ILS spliceosome,^17^ where the U2, U5 and U6 snRNAs together with the splicing proteins need to be recycled for subsequent assembly on the next intron, while the intron-lariat can be processed or degraded.

Besides the eight conserved helicases driving splicing in all eukaryotic cells,^18^ higher eukaryotes express five additional helicases involved in the splicing mechanism: DDX42, Abstrakt, DHX35, eIF4A3 and Aquarius (AQR).^19^ In a recent study, AQR was reported to aid DHX16 in the catalytic activation of the spliceosome.^20^ Meanwhile, evidence has been provided that DHX35, in cooperation with the G-patch protein GPATCH1, plays a role in the quality control of suboptimal pre-mRNAs splicing in *Schizosaccharomyces pombe* and *Cryptococcus neoformans*.^21–24^ In *C. neoformans*, DHX35 and GPATCH1 associate with active spliceosomes, where they suppress splicing of substrates with weak 5’ss and 3’ss, particularly weak intron sequences.^24^ Both factors were also found in catalytically active human spliceosomes,^25,26^ indicating that their function is conserved from fungi to human cells. However, what the substrate of a DHX35–GPATCH1 complex might remain poorly understood. Additionally, different G-patch proteins are setting the stage for their cognate RNA helicases by facilitating their specific recruitment to the spliceosome and enhancing their ATPase activity.^27^ For example, *sc*Prp2 and its cofactor *sc*Spp2 promote the transition from B^act^ to B*,^28,29^ while the G-patch factor *sc*Ntr1, together with *sc*Ntr2, activates *sc*Prp43, which then disassembles the ILS spliceosome.^30,31^ Recently, cryo-EM analysis of *Caenorhabditis elegans* (*C. elegans* or *ce*) and human ILS spliceosomes revealed how the G-patch factor TFIP11 (*sc*Ntr1) and PAXBP1 (*sc*Ntr2) identify the terminal ILS spliceosome and guide DHX15 to its substrate, the 3′-end of U6 snRNA.^32,33^ DHX15 has also been identified in numerous spliceosomal assembly stages throughout the splicing cycle,^34^ where it is suggested to function in disassembling aberrant spliceosomes.^35^ In human cells, the G-patch factor SUGP1 has been reported to activate DHX15 during quality control of early spliceosomes before the formation of the U2/BS.^36–38^ Moreover, the G-patch proteins RBM5 and RBM10 appear to play a role in regulating splicing after recognition of the BS,^39^ and RBM5 has been implicated in DHX15 activation in early spliceosomes.^40^ These spliceosome intermediates contain suboptimal pre-mRNAs, that comprise aberrant BSs or non-canonical 5’ss or 3’ss, and which thus have been rejected by the proofreading activity of other RNA helicases.^36,41,42^

Despite the plethora of high-resolution cryo-EM structures revealing spliceosomes in the functional states (see above), the structural basis of the recognition of aberrant pre-mRNAs and the disassembly of the resulting aberrantly assembled spliceosomes remain poorly understood. Here, we provide high-resolution cryo-EM structures of such so far undetected spliceosomal intermediates that contain suboptimal splicing substrates, isolated via the RNA helicase DHX15 from the thermophilic eukaryote *Chaetomium thermophilum* (*C. thermophilum* or *ct*). Our study identifies the RNA helicase DHX35 and its G-patch activator GPATCH1 as key players in the recognition of erroneous splicing processes involving a suboptimal 5’ss, which contains a bulged loop. We show that U2 snRNA is the substrate of DHX35, which has dissociated the U2/BS helix. Additionally, DHX15 is placed close to its substrate, the U6 snRNA 3'-end. Our findings thus provide structural insights into how spliceosomal complexes containing an aberrant pre-mRNA are recognized and primed for disassembly.

## Results

### Isolation of spliceosomes from a eukaryotic thermophile

*C. thermophilum* is a thermophilic filamentous fungus that optimally grows at 50 °C and can propagate at temperatures up to 60 °C. In the past, a number of conserved macromolecular RNA–protein complexes (e.g., pre-ribosomes) have been successfully isolated from *C. thermophilum*, which turned out to be very suitable for biochemical and structural analyses.^43^ Therefore, we wanted to analyze the spliceosome from *C. thermophilum*. We have created a list of human and baker’s yeast splicing factors and assigned *C. thermophilum* homologs using the annotation of several databases (see “Materials and methods”). Notably, the genome of *C. thermophilum* encodes nearly all homologs of the human spliceosomal proteome, many of which are absent in the baker’s yeast, another classical model for spliceosome functional studies in the past (Supplementary information, Table S1). However, baker’s yeast exhibits comparatively few introns throughout its genome and hence has lost many splicing factors present in human cells.^24^ In fact, the inventory of spliceosomal proteins from *C. thermophilum* that we annotated in this study is surprisingly more similar to the composition of human than baker’s yeast spliceosomes (Supplementary information, Table S1), consistent with the finding that not only a large number of *C. thermophilum* gene transcripts are spliced, but a significant portion of them are subject to alternative splicing.^44^

In order to isolate *C. thermophilum* spliceosomes captured in different disassembly and/or quality control states, we used the conserved disassembly factor DHX15 (Prp43 in baker’s yeast; in the following, we will use the names of the human splicing factors) as a bait, as it has been suggested to be involved in ‘discard pathways’ of rejected intron-containing pre-mRNAs.^35–38,40–42^ To initiate the purification, we transformed *C. thermophilum* protoplasts with a gene construct that encodes N-terminally tagged DHX15 under the control of a constitutive (*actin*) promoter as previously described.^45,46^ Subsequent tandem-affinity purification of tagged DHX15 revealed co-precipitation of numerous spliceosomal proteins, including spliceosome core proteins (e.g., U2 snRNP, U5 snRNP, NTC and NTR proteins) and known disassembly factors (TFIP11, GCFC2, CWF19L1, CWF19L2) (Supplementary information, Fig. S1a and Table S2). In line with DHX15’s role in ribosome biogenesis, we find proteins involved in ribosome assembly, albeit in lower amounts, indicating that in *C. thermophilum*, DHX15 is preferentially associated with spliceosomal complexes. Moreover, spliceosomal proteins involved in catalytic spliceosomes, such as IBC or C* factors, were co-precipitated. Notably, among the C* factors, DHX35, WDR83 and GPATCH1, which were described as a functional unit in *S. pombe*,^23^ were the most abundant.

Prompted by these results, we collected cryo-EM data from the DHX15 sample, which revealed three major spliceosome complexes (Supplementary information, Fig. S1b). One state corresponds to the ILS complex, while the other two complexes represent aberrant spliceosomal assembly states stalled prior to step I catalysis and subjected to quality control. We have thus termed them quality control complexes B*^Q1^ and B*^Q2^, as they represent B^act^ complexes that have been intercepted by quality control while on their way to B*.

### Cryo-EM structure of the *ct*ILS complex

**Fig. 1:**
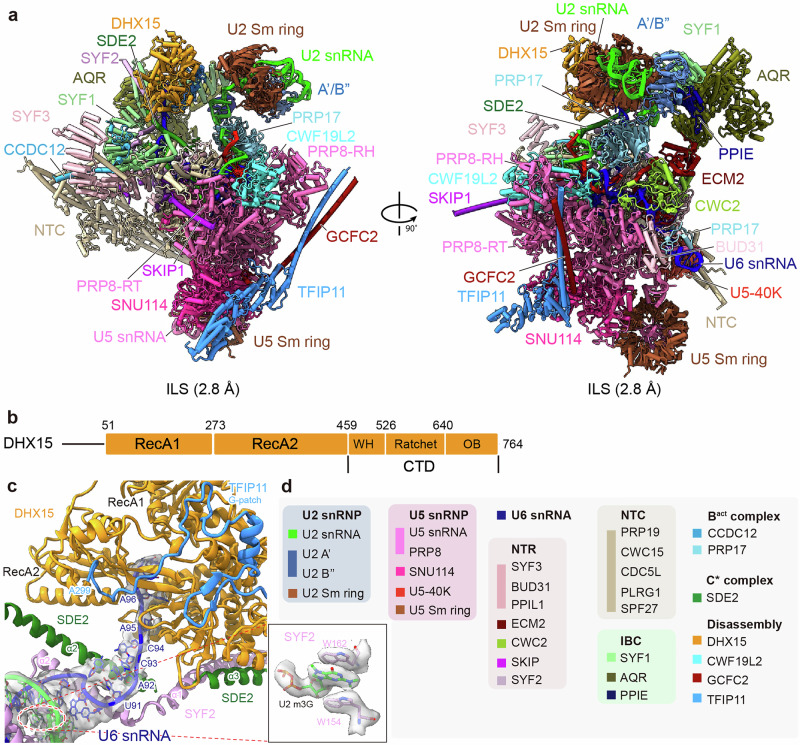
Cryo-EM structures of the *C. thermophilum* ILS complexes. **a** Cryo-EM structure of the ILS state of *C. thermophilum* observed after affinity purification of DHX15. Two different views of the molecular model of the *ct*ILS state are presented, with average resolution expressed in Å. **b** A schematic of the domain architecture of DHX15. **c** In the *ct*ILS state, the resolution as illustrated with the density map of the U6 snRNA allows the identification of the 3′-end of the U6 snRNA, which protrudes into the RNA binding tunnel of DHX15. The density of the 5’ m3G cap of the U2 snRNA is shown as an insert (down right). The density map is derived from a focused refinement map. **d** Summary of all modeled proteins from the *ct*ILS structure and RNAs with color code.

We could resolve the *ct*ILS structure at an average resolution of 2.8 Å, with the variable local resolution, ranging from 2.2 Å in the core region to ~10 Å in the periphery (Fig. 1; Supplementary information, Figs. S2, S3 and Table S3). This data allowed us to use a combinatorial approach, where we either de novo built the core (e.g., PRP8) or fitted AlphaFold-predicted^47^ models into the peripheral densities (e.g., DHX15, TFIP11, GCFC2 (*sc*Ntr2)). Together, we were able to obtain a near-complete molecular model of the *ct*ILS complex (Fig. 1a; Supplementary information, Fig. S3 and Table S3) with an excellent resolution in the spliceosome core, highlighting *C. thermophilum* as a very promising organism for determining spliceosome structures.

In this state, we could clearly trace the U2, U5, and U6 snRNAs, as well as the intron-lariat RNA (Supplementary information, Fig. S3b). The absence of mature mRNA within the U5 region confirmed that this is the terminal ILS complex. It is highly similar to the recently described *C. elegans ce*ILS” (PDB:8RO1) complex^32^ with a similar overall architecture and nearly identical composition (Fig. 1d; Supplementary information, Fig. S4a and Table S4), with the exception of the CWC2–ECM2 complex, and CWF19L1 that we could not unambiguously identify in our structure due to the low local resolution (Supplementary information, Fig. S4).

Additionally, we could unambiguously position the DHX15–TFIP11–GCFC2 complex, which is responsible for disassembling the ILS complex in the following step (Fig. 1a–c). As observed before, TFIP11 is associated with SNU114 and PRP8 via its helical bundle and forms a coiled-coil interaction with GCFC2 via its long α-helix^32^ (Fig. 1a). However, we cannot observe the helical repeat GCFC domain of GCFC2 (Supplementary Fig. S4a). DHX15 is a DEAH-box RNA helicase that unwinds its substrate RNA with a 3′–5′ processivity and contains two RecA domains and a C-terminal domain comprising Wing Helix (WH), Ratchet and OB domains (Fig. 1b). Similar to previous observations in the metazoan structure, in the *ct*ILS structure, we found the DHX15–TFIP11 (G-patch domain) complex docked on a surface provided by SYF1, SYF2 and SDE2 (Supplementary information, Figs. S4a, S5a). It engages the 3’-end poly-U stretch of the U6 snRNA as substrate, which emerges from the U2/U6 helix II into the RNA binding tunnel of DHX15 (Fig. 1c; Supplementary information, Figs. S3c, S5a). Similar to the *ce*ILS” structure, this region is stabilized by SYF2 and SDE2 and guided towards the active site of DHX15, where alpha-helices of both proteins wrap around the U2/U6 helix II, while SYF2 wedge-helix (α2 helix) binds into the slightly opened U2/U6 helix II (Fig. 1c; Supplementary information, Fig. S5a). Our structure also allows the identification of the 5′ terminal m_3_G cap structure^48,49^ of the U2 snRNA (Fig. 1c), which is stacked in between W154 and W162 of SYF2. DHX15 is observed in a closed conformation, resembling the ATP-free form of previously reported RNA-bound DHX37 (Supplemental Fig. S5b). Thus, DHX15 appears to await ATP binding in a closed apo-state, ready to unwind the U6 snRNA by exerting its pulling force by successive cycles of ATP hydrolysis.

Taken together, our *ct*ILS model shows a high compositional and structural similarity with the recently published ILS structures, suggesting a high degree of conservation of spliceosomal structures among *C. thermophilum, C. elegans*, and higher eukaryotes. Moreover, our data support the idea that DHX15-purified *C. thermophilum* spliceosomes are physiological.

### Quality control of the aberrant B* spliceosome by DHX35–GPATCH1

**Fig. 2:**
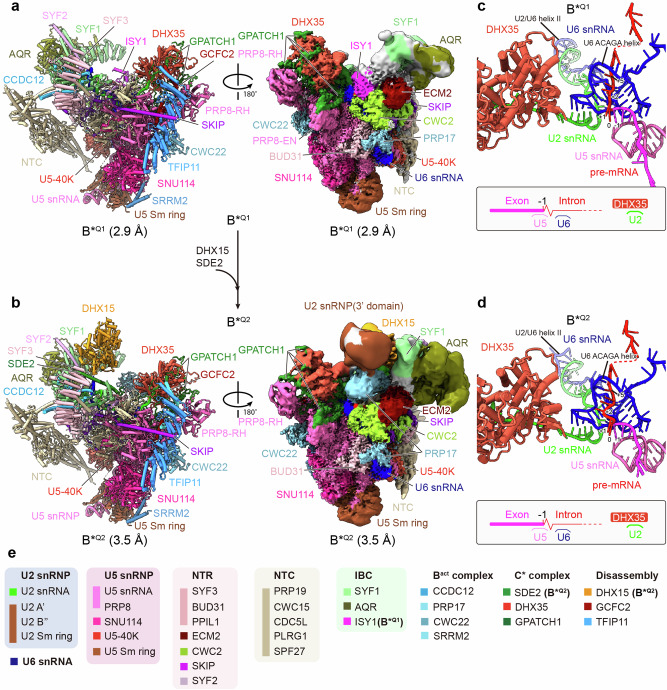
Cryo-EM structures of the *C. thermophilum* B*^Q^ complexes. **a**, **b** Two different views of the molecular models (left) and cryo-EM density maps (right) of the *C. thermophilum* spliceosome in B*^Q1^ (**a**) and B*^Q2^ (**b**). The compositional change during the transition is indicated in the middle. The composite cryo-EM density maps were generated from multi-body refined maps. The IBC module is shown at different contour levels. **c**, **d** The RNA structures in B*^Q1^ (**c**) and B*^Q2^ (**d**) are shown together with DHX35 (tomato red), which is bound to the U2 snRNA (green). The suboptimal pre-mRNA in the active center is highlighted. A simplified scheme of the RNA interactions is illustrated in the bottom row, with the 5’ ss bulge indicated as a zigzag line. **e** Summary of all modeled proteins from the B*^Q^ structures and RNAs with color code.

In addition to the *ct*ILS state, cryo-EM revealed two novel spliceosome states, refined to a molecular resolution of 2.9 Å and 3.5 Å, respectively (Fig. 2; Supplementary information, Figs. S1b, S2, S6 and Table S3). The two complexes contain the snRNAs U2, U5 and U6 and a fully assembled catalytic U2/U6 RNA active site, which is docked into the central cavity of the closed PRP8 structure and stabilized by NTC and NTR proteins, as well as IBC and IBC related proteins (Fig. 2**;** Supplementary information, Table S4). The absence of U2 SF3 A and SF3 B proteins, the Retention and Splicing (RES) protein complex and B^act^ proteins RNF113A and CWC27, indicate that the spliceosomes have passed the B^act^ state and have been catalytically activated by the combined action of PRP2 and AQR helicases^20^ (Fig. 2; Supplementary information, Table S4). As C complex proteins such as YJU2 or CWC25 or C* specific proteins are largely absent (except for SDE2, see below), and the pre-mRNA contains an intact 5′ss and has thus not yet undergone first step catalytic cleavage (Fig. 2c, d), we conclude that the isolated spliceosomes have been intercepted/stalled at a catalytically activated B* assembly stage. Interestingly, both spliceosomes contain the RNA helicase DHX35 in complex with the G-patch protein GPATCH1 (Fig. 2).

DHX35 RNA helicase and GPATCH1 have been shown to be involved in the quality control of pre-mRNA splicing containing, among others, suboptimal 5′ss sequences.^22–24^ Indeed, close inspection of the conformation of the pre-mRNA 5′ss region reveals the likely reason, why first-step cleavage at the 5’ss may be hindered. While the base pairing interaction between the U5 snRNA loop I and the 3′-end of the upstream exon is well resolved, the RNA helix between the U6 ACAGA box and the 5’-end of the intron is only poorly defined, which could be due either to a higher degree of flexibility, or due to the fact that we likely average the structures of spliceosomes containing distinct pre-mRNA molecules (Supplementary information, Fig. S7a). Due to the flexibility/instability of the U6 ACAGA helix in the quality control states, an unambiguous assignment of the bases was not possible, and we used the RNA structure from the *ct*ILS state as a reference for tentative orientation (Supplementary information, Fig. S7a). We would like to emphasize, however, that we were able to track the RNA backbone and thus clearly see that the pre-mRNA bulges out into the active site center between the U5/5’-exon and U6 ACAGA helices. Normally the two nucleotides G^+1^ and U^+2^ are positioned between the duplexes, but the distance in the B*^Q^ complexes is spanned by three bases. (Supplementary information, Fig. S7b). Consistent with this, DHX35 and GPATCH1 have been reported to regulate the aberrant splicing of 5’ss within pre-mRNAs that contain introns with non-appropriate nucleotides at positions 4, 5 and 6 (in fungi, positions 5 and 6 should ideally be “GU”) in *C. neoformans*,^24^ leading to weak base-pairing with the ACAGA box of the U6 snRNA. To analyze whether DHX35–GPATCH1 have a similar role in higher eukaryotes, we used the dTAG system to knock down DHX35 and GPATCH1 in a mouse cell line^50^ (Supplementary information, Fig. S7c). RNA sequencing analysis was performed to examine splicing events, as described previously.^24^ Our results revealed that, similar to *C. neoformans*, splicing events of pre-mRNAs containing less effective 5′ss motifs enriched after DHX35 and GPATCH1 knockdown (Supplementary information, Fig. S7d, e). These findings are consistent with the idea, raised above, that non-canonical base-pairing may thus lead to a misalignment between the U6 ACAGA box and the 5′-end of the intron (positions 4–6), eventually resulting in a 5′ss bulge.

In optimal splicing substrates, the U5/exon and U6/intron base pairing defines and positions the 5′ss for the step I catalysis reaction, thus contributing to the efficiency of the first trans-esterification reaction.^51–56^ During the B^act^-to-C transition, the U2/BS helix (U2 snRNA base pairs with the BS forming an RNA helix) is relocated (Supplementary information, Fig. S8a), causing the BS-adenosine to form a base triplet with an intron uridine and U2 snRNA, while it is further stabilized by partially stacking with the G^+1^ of the 5′ss^57,58^ (Supplementary information, Fig. S8b). This rearrangement positions the BS-adenosine 2'-OH group optimally for a nucleophilic attack on the 5′ss phosphate group (Supplementary information, Fig. S8b). However, the U2/BS helix relocation is prevented in the *ct*B*^Q^ complexes, as the 5′ss bulge prevents the accommodation of the BS-adenosine, suggesting that the suboptimal conformation of the pre-mRNA hinders further progression during the splicing reaction (Supplementary information, Fig. S8b). Moreover, the U2/BS helix is dissociated in our structure (see below) (Fig. 2c, d; Supplementary information, Fig. S8c). This premature helix dissociation further implicates the aberrant conformation of the pre-mRNA as a basis for the resulting quality control processes. We conclude that we have captured a spliceosome, which contains an aberrant 5’ss conformation and is subjected to quality control by DHX35 and GPATCH1.

### Quality control of the aberrant B* spliceosome by DHX15–TFIP11

In addition to a pre-mRNA containing a 5′ss bulge, we found disassembly factors TFIP11 and GCFC2 in both quality control complexes, and DHX15 as well as DHX15 docking protein SDE2 in the *ct*B*^Q2^ structure (Fig. 2a, b). While the local resolution of the TFIP11–GCFC2 region in the *ct*B*^Q^ structures is low, however, *sc*Ntr1 (TFIP11) is known to bind at this position by interacting with *sc*Prp45 (SKIP) in both *S. cerevisiae* and *S. pombe*.^33,59^ Moreover, the focus-refined density in state B*^Q1^ closely matches the expected shape of TFIP11 (Supplementary information, Fig. S6b). Additionally, with sufficient resolution, we can fit with confidence GCFC2’s α-helix (aa 343–367) into the density element adjacent to TFIP11 (Supplementary information, Fig. S6b). Finally, we purified TFIP11 and GCFC2 from *C. thermophilum* extracts to verify their association with spliceosomes containing the GPATCH1–DHX35 complex. Mass Spectrometry analysis confirmed the presence of GPATCH1, DHX35, and WDR83 in both samples (Supplementary information, Fig. S9a and Table S2), which is in line with previous findings from *S. pombe* showing that *sp*Ntr1 and *sp*Ntr2 associate with *sp*Gpl1–Gih35–Wdr83.^21^ Reciprocal purifications of DHX35 and WDR83 further reinforce this finding, as both proteins pull down spliceosomes containing the disassembly DHX15–TFIP11–GCFC2 complex, and other splicing factors found in catalytically active spliceosomes (Supplementary information, Fig. S9a and Table S2).

Since the DHX15–TFIP11–GCFC2 complex is known to associate with ILS spliceosomes primed for disassembly,^32^ *ct*B*^Q2^ might represent an intermediate nearing disassembly, while *ct*B*^Q1^ could represent its precursor. Within the *ct*B*^Q2^ complex, DHX15 occupies a binding site similar to that in the *ct*ILS complex, interacting with SYF2 and SDE2 (Figs. 1a, 2b; Supplementary information, Fig. S5a), which is normally recruited to the C complex after step I splicing reaction has occurred.^26^ While in the *ct*ILS spliceosome, TFIP11–GCFC2 binds to a surface provided by PRP8-RT and SNU114, in the *ct*B*^Q^ states, this complex binds at a different region **(**Figs. 1a, 2a; Supplementary information, Fig. S10a, b). Specifically, the TFIP11–GCFC2 dimer interacts with SKIP (*sc*Prp45), while GCFC2 directly binds to PRP8, blocking the recruitment of the RNA helicases DHX16 (*sc*Prp2), DHX38 (*sc*Prp16) and DHX8 (*sc*Prp22), all of which are needed for further splicing progression (Supplementary information, Fig. S10c, d). Meanwhile, the binding interface that is used by the TFIP11–GCFC2 complex in the *ct*ILS complex is occupied in the B*^Q^ states by SRRM2 and CWC22 (Supplementary information, Fig. S10b), two proteins that are recruited to the B^act^ complex to help to stabilize the 5′ exon.^60^ This suggests that the DHX15–TFIP11–GCFC2 complex is recruited in the *ct*B*^Q^ particles for quality control purposes and to disassemble the spliceosome with the pre-mRNA still present, which is in contrast to the terminal ILS complex, where the pre-mRNA has been already released.

In summary, the dual presence of splicing factors involved in catalysis and spliceosome disassembly, together with an unspliced pre-mRNA, indicates that the novel *ct*B*^Q^ states apparently are catalytic spliceosomes that are in the process of recognizing (a) suboptimal pre-mRNA(s) and hence might be primed for disassembly.

### GPATCH1 recognizes the aberrant 5′ss bulge loop

**Fig. 3:**
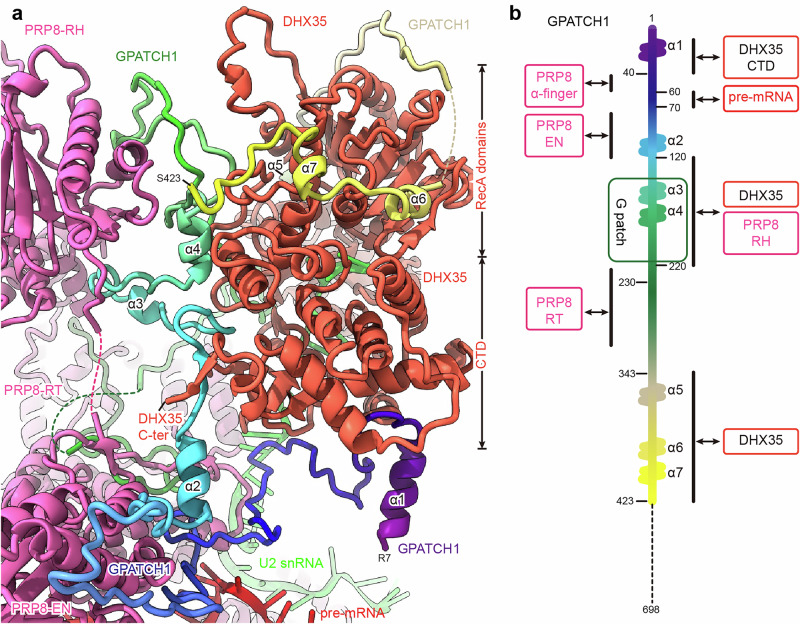
GPATCH1 extensively interacts with PRP8 and DHX35. **a** Overview of key interaction sites between GPATCH1 (rainbow color, from purple to yellow), PRP8 (pink) and DHX35 (tomato red). GPATCH1 (rainbow) binds to multiple PRP8 domains, including the EN, RT and RH domains, and to the DHX35 helicase. Unstructured regions of PRP8 and DHX35 are indicated by dashed lines. **b** Schematic representation of GPATCH1 interactions. Key interaction sites along the GPATCH1 sequence are highlighted.

**Fig. 4:**
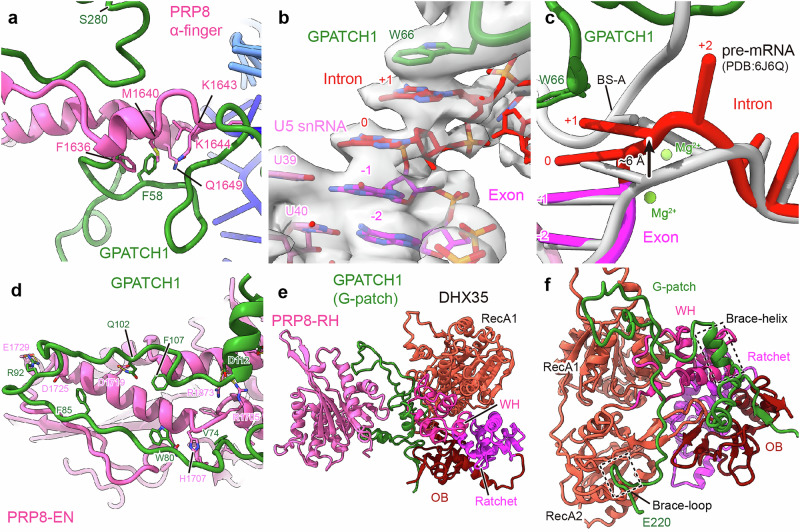
GPATCH1 recognizes stalled splicing intermediates and anchors DHX35 to the spliceosome. **a** GPATCH1 region aa 40–60 binds to the α-finger of PRP8. **b** W66 of GPATCH1 probes the active center downstream of the –1 position of the pre-mRNA. The density map is taken from a focused refinement map. **c** Comparison of the pre-mRNA in B*^Q1^ with that in the B* complex (PDB: 6J6Q). The proposed G^+1^ nucleotide of the 5’ss is about 6 Å away from the active cleavage site. The active Mg^2+^ shown are taken from 6J6Q. The movement of the G^+1^ nucleotide is indicated by the black arrow. **d** GPATCH1 region aa 70–120 binds to the EN domain of PRP8. **e** GPATCH1 (aa 120–220) is sandwiched between the PRP8 RH domain and DHX35. **f** The G-patch domain (aa 152–220) of GPATCH1 binds to DHX35, which is depicted with color-coded domains (RecA domains, tomato red; WH domain, deep pink; Ratchet, magenta; OB-fold domain, dark red).

GPATCH1 is a G-patch domain-containing protein with a highly conserved N-terminus containing the G-patch domain (aa 152–220) (Supplementary information, Figs. S11, S12). While functional studies indicated that GPATCH1 is the activating G-patch protein for DHX35,^23^ the structural basis for this complex formation and its interface with the spliceosome remained unclear. We were able to model the N-terminal part of GPATCH1 in our structure, including the functionally crucial G-patch domain (Figs. 3, 4; Supplementary information, Figs. S6, S13). Essentially, GPATCH1 binds to the spliceosome via PRP8, probes the pre-mRNA within the active site center, and anchors DHX35 to the spliceosome. In doing so, GPATCH1 embraces DHX35 by multiple interfaces in addition to its GPATCH domain (Fig. 3). At the same time, it binds to multiple strategically important regions of PRP8, including the PRP8-EN, Prp8 α-finger and PRP8-RH (Fig. 3). Additionally, GPATCH1 contacts the U2 RNA as well as the aberrant 5’ss bulge, suggesting that it plays important roles beyond tethering DHX35 to PRP8 and activating its ATPase activity (Fig. 3).

Consistent with the crystal structure of DHX15 in complex with the G-patch motif of NKRF^61^ or SUGP1,^62^ the brace-helix and brace-loop of GPATCH1’s G-patch motif bind to DHX35’s WH and RecA2 domains, respectively (Fig. 4f; Supplementary information, Fig. S13c). The remaining stretch of the G-patch motif runs across the backside of the RNA binding channel of DHX35 towards the brace loop (Fig. 4f). Thus, the G-patch motif effectively tethers the WH and RecA2 domains together, which is important for efficient RNA helix unwinding activities of DHX helicases.^61^ Moreover, the GPATCH1 region comprised of the amino acids 360–438 wraps around the outer regions of DHX35’s RecA1 and RecA2 domains, further stabilizing the catalytically important domains (Supplementary information, Fig. S13d).

Both conserved stretches flanking the G-patch domain of GPATCH1 (aa 70–120 and aa 220–298) form a large interaction surface with the PRP8-RT/Linker/EN domains (Fig. 4d; Supplementary information, Fig. S13e). The region of GPATCH1 (aa 143–150) immediately adjacent to the brace-helix on the N-terminal side forms an α-helix that interacts with the PRP8-RH domain (Supplementary information, Fig. S13f). Additionally, the G-patch domain of GPATCH1 is significantly longer than other G-patch motifs (Supplementary information, Fig. S11b), with an elongated loop that forms a substantial interface with the back side of the PRP8-RH domain (Fig. 3a; Supplementary information, Fig. S13f). As a result, the G-patch domain is sandwiched between PRP8 and DHX35 (Fig. 4e). This places the PRP8-RH domain in a novel position that is only observed in the B*^Q^ complex, where it is close to the PRP8 Linker domain (Supplementary information, Fig. S13g). Since the PRP8-RH domain is a platform recruiting different splicing factors throughout the splicing cycle and key for the successful docking of the extended U2/BS helix during its transformation from B^act^ to the B*/C position, it is plausible to assume that in the presence of GPATCH1, the extended U2/BS helix will remain flexible.

Following an α-helical linker region, the upstream region of GPATCH1 (aa 65–109) forms a long loop, which covers almost the entire upper part of the PRP8-EN domain, stabilized by several hydrophobic interactions and hydrogen bonds (Fig. 4d; Supplementary information, Fig. S13h–k). It then runs along the C-terminal α-helix of the α-finger (Fig. 4a), locking it in a conformation similar to the B^act^ complex. The PRP8-RH domain and the PRP8 α-finger have to undergo significant rearrangements during the B^act^-to-C transition in order to create a docking site for the U2/BS helix^57^ (Supplementary information, Fig. S13l, m). Consequently, by interacting with the PRP8 α-finger, GPATCH1 blocks the movement of U2/BS helix, while at the same time positioning itself to probe and stabilize the conformation of the 5’ss bulge in the active center via its aromatic W66 by contributing to the stacking interaction formed by the 5’ss nucleotides (Fig. 4b). In this conformation, the supposed G^+1^ of the 5’ss is displaced about 6 Å in distance from the active site center when compared to state B* (PDB: 6J6Q) (Fig. 4c). Notably, at this position, GPATCH1 also spatially clashes with the position of the 3’ss during the second cleavage step (Supplementary information, Fig. S13n), thereby preventing further progression in the splicing cycle. Moreover, the binding of DHX35–GPATCH1 is incompatible with the binding of the step I factors YJU2 and CWC25 (Supplementary information, Fig. S13b), which are crucial for efficient step I catalysis as they stabilize the U2/BS helix, with CWC25 suggested to push the U2/BS helix into the active site center.^57,63^

Taken together, our data reveal that GPATCH1 via its N-terminal region recognizes stalled spliceosomes containing a suboptimal splicing substrate and ensures quality control by anchoring DHX35. The docking of GPATCH1 on crucial PRP8-RH/α-finger/EN domains blocks the recruitment of step I factors and impedes the crucial relocation of the U2/BS helix, therefore preventing further progression in the splicing reaction. Thus, it is likely that the interaction of the N-terminal region of GPATCH1 with PRP8-EN and PRP8 α-finger is the key event, initiating the quality control process of the aberrantly assembled spliceosome. The high conservation of the N-terminus of GPATCH1 supports the idea that GPATCH1–DHX35 is likely bound to PRP8 in a similar manner in different organisms.

### DHX35 unwinds the U2/BS helix in the B*^Q^ spliceosome priming it for disassembly

**Fig. 5:**
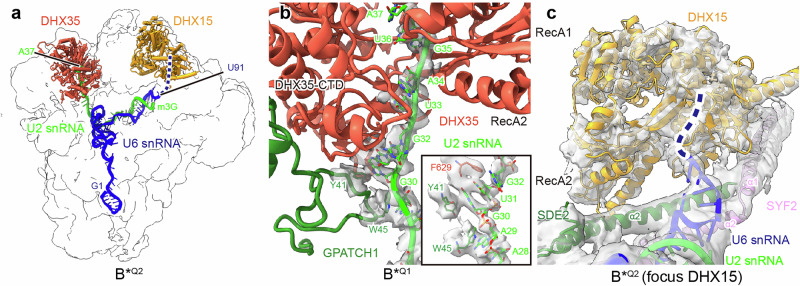
DHX35 and DHX15 prime the stalled spliceosome for disassembly. **a** Illustration of the position of the DHX35 (tomato red) and DHX15 (orange) helicases within the B*^Q2^ complex, including the depiction of the U2 snRNA (green) that threads into DHX35 (tomato red) and the U6 snRNA (blue) located close to DHX15 (orange). The density map of the B*^Q2^ complex is shown in the background, with the predicted binding path of U6 snRNA in DHX15 indicated by a dashed line. **b** DHX35 binds to the region of the U2 snRNA that forms a helix with the BS of the pre-mRNA in catalytic spliceosomes. The density map for the U2 snRNA is shown to illustrate the resolution and connectivity. Detailed interaction of GPATCH1 with U2 snRNA is shown with a density map as an insert. The molecular model and density map are derived from B*^Q1^. **c** Close-up view displaying the rigid body fit of DHX15 within the focused refined cryo-EM map in B*^Q2^ (focus DHX15). The predicted binding path of U6 snRNA in DHX15 is indicated by a dashed line.

In the *ct*B*^Q2^ complex, we could unambiguously assign both DHX35 and DHX15 helicases, with DHX35 at atomic resolution (Fig. 5a; Supplementary information, Fig. S6a, b). DHX35 RNA helicase has been shown to be involved in the quality control of pre-mRNA splicing with, among others, suboptimal 5′ss sequences.^24^ However, the precise substrate of DHX35 has remained unclear.

Strikingly, in *ct*B*^Q^ complexes, DHX35 is bound to the single-stranded BS region of U2 snRNA, comprising nucleotides G32 to A37, within the RNA binding channel of its RecA domains. This is aided by GPATCH1, which forms two key stacking interactions with the U2 snRNA (amino acid W45 with RNA base A29, and amino acid Y41 with RNA base G30), guiding the U2 snRNA into the RNA binding of DHX35 (Fig. 5b). DHX35 is observed in an RNA-bound closed conformation, but in a nucleotide free state, similar to the apo-form of previously reported RNA-bound DHX37 (Supplementary information, Fig. S14a).^64^ The docking of a single-stranded U2 BS region at the RecA domains of DHX35 strongly suggests that we have captured the helicase in a state following dissociation of the extended U2/BS helix. Consistently, DHX35 has adopted a position in B*^Q^ which is mutually exclusive with the position of the U2/BS helix in a C or ILS complex, where splicing factors YJU2 and CWC25 stabilize the U2/BS helix (Supplementary information, Fig. S14b), indicating that DHX35 has a crucial role in preventing further progression in splicing catalysis.

DHX15 is fitted rigidly due to lower local resolution and adopts a similar conformation as observed in the *ct*ILS complex (Fig. 5c; Supplementary information, Fig. S14c). The binding site of DHX15 does not differ between *ct*ILS and B*^Q2^, and between species, highlighting the high conservation of the disassembly mechanism by DHX15. In the *ct*B*^Q2^ state, DHX15 is positioned in the vicinity of the U6 snRNA (Fig. 5c; Supplementary information, Fig. S14c). Although we cannot unambiguously place the 3′-end of U6 snRNA in the RNA binding tunnel of DHX15, a comparison of DHX15 and U6 snRNA between the *ct*B*^Q2^, *ct*ILS and *ce*ILS states raises the possibility that DHX15 could be positioned for spliceosome disassembly by unwinding U6 snRNA (Fig. 5c; Supplementary information, Fig. S14d).

Taken together, our structures show that both DHX35 and DHX15 engage with spliceosomal quality control states. We identify the U2 snRNA as target of DHX35, which has dissociated the critical U2/BS helix, while DHX15 binds near U6 snRNA, likely preparing the complex for disassembly. An important implication of our findings is that the disassembly of quality control spliceosome complexes is driven by two DEAH-box RNA helicases.

## Discussion

The need to excise introns with single nucleotide precision requires mechanisms that establish splicing fidelity by proofreading and discarding suboptimal splicing substrates. Our study uncovered the structural details of how suboptimal 5’ss can be recognized and subsequently disassembled by the specific recruitment of two DEAH-box RNA helicases, DHX35 and DHX15. We found that GPATCH1, the activator of DHX35, plays a crucial role in the recognition of aberrant splicing substrate conformations. Moreover, it specifically anchors DHX35, which then can engage with its target, the U2 snRNA, and dissociate the U2/BS helix. DHX15 on the other hand is recruited to an interaction surface similar to the *ct*ILS state and close to its target, the U6 snRNA. In the B*^Q^ structures, TFIP11 and GCFC2 occupy a different region compared to the ILS state, raising the question whether TFIP11 can activate DHX15, whether further rearrangements are needed or whether DHX35 can dissociate the U2/U6 helix on its own. Interestingly, both of these positions have been observed before.^32^ The position occupied in the B*^Q^ complex is similar to that seen in the baker’s yeast ILS complex,^33^ suggesting that both locations could be binding sites used to activate DHX15. In baker’s yeast, the NTR complex (comprising *sc*Ntr1 (*ct*TFIP11), *sc*Ntr2 (*ct*GCFC2) and *sc*Prp43 (*ct*DHX15)) can disassemble spliceosomes in vitro at different stages after the specific action of proofreading RNA helicases *sc*Prp2 (*ct*DHX16), *sc*Prp16 (*ct*DHX38) and *sc*Prp22 (*ct*DHX8),^65^ which are crucial for branch-formation (B^act^ to B*), exon joining (C to C*) and mRNA release (P to ILS), respectively. Notably, in some spliceosomal complexes, stable NTR binding was required but not sufficient for spliceosome disassembly, indicating that rearrangements upon NTR binding might be necessary to reach a 'disassembly competent' stage.^65^ Similarly, the position in B*^Q^ may represent different stages in the DHX15 activation process, raising the possibility that further rearrangements in B*^Q^ are necessary to allow binding of TFIP11 to the position it adopts in the ILS complex.

**Fig. 6:**
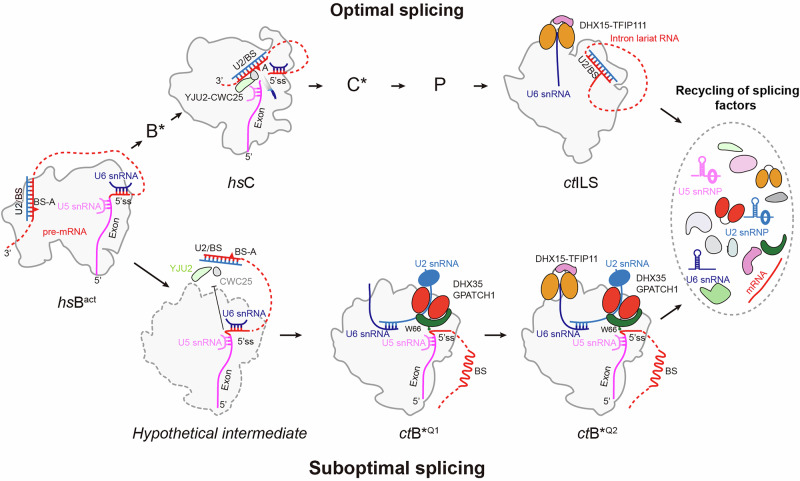
Structural model of the quality control of suboptimal splicing substrates involving DHX35 and DHX15. Schematic of rearrangements and repositioning of key factors during optimal splicing processes and disassembly by DHX15 (top) and during suboptimal splicing processes (bottom) leading to stalled spliceosomes, quality control and disassembly by DHX35 and DHX15.

Previous studies have detailed the structural rearrangements that promote catalysis of the first splicing reaction.^57,66,67^ After catalytic activation and as part of the transition from the B^act^ to the C complex, the U2/BS helix is translocated a large distance from its position in the B^act^ state to the catalytic center in the C state.^20,57^ This relocation is facilitated by the rearrangement of the PRP8 RT/EN and RH domains, which provide a binding surface for the U2/BS helix close to the catalytic center. We propose that U2 snRNA translocation is prevented when the catalytic center contains a 5’ss bulge and hence, an altered conformation (Fig. 6), possibly due to inefficient recognition of the 5’ss by the U6 snRNA.

In the B*^Q^ states, the pre-mRNA appears to contain a 5'ss with a bulged-out loop and increased flexibility around the U6 ACAGA/intron helix. The 5'ss bulge and the concomitant position of the 5'ss phosphate group, now distant from the catalytic Mg^2+^ ions, could hinder an efficient docking of the U2 snRNA as well as the BS-adenosine. As a consequence, the U2 snRNA and the U2/BS helix could oscillate back and forth. In this situation, the PRP8-RT/EN and PRP8-RH domains remain accessible, which offers GPATCH1 a docking surface to position DHX35 for binding and engaging the U2 snRNA, and, at the same time, prevent further progression in the splicing reaction. In the B^act^ state, the SF3A and SF3B complexes keep the 3’-end of the U2 snRNA in a distinct stable position.^67,68^ During the B^act^-to-B* transition, they are removed to facilitate the U2/BS translocation.^20,29^ Therefore, the dislodgment of these two complexes in B*^Q^ further destabilizes and exposes the U2 snRNA. Additionally, the U2 snRNA switches between SLIIa, SLIIb and SLIIb/c,^57,69,70^ and thus it is conceivable that the resulting dynamic situation could produce single-stranded U2 snRNA nucleotides downstream of the U2/BS helix. DHX35 then could bind U2 snRNA and unwind the U2/BS helix by moving in a 3’ to 5’ direction.

Similar to state *ct*ILS, DHX15 in state B*^Q2^ is positioned close to the U2/U6 helix II and may access the 3’-end of the U6 snRNA, ready to disassemble the entire spliceosome. Such a mechanism is consistent with previous studies implicating DHX15 in the disassembly of the spliceosome that has been discarded from the splicing cycle at different stages.^36,41,65^ Recently, cooperativity between two RNA helicases, PRP2 (DHX16) and AQR, was described during the crucial spliceosome catalytic activation.^20^ PRP2 first translocates along the intron RNA, opening the B^act^ complex, while AQR subsequently facilitates the relocation of the U2/BS helix to the catalytic center and promotes the dissociation of SF3A and SF3B complexes. The coordination of these two helicases opens a window for proofreading, allowing the spliceosome to recognize and discard suboptimal pre-mRNAs that are similar to those observed in our study. DHX35 and DHX15 may then drive the disassembly of rejected spliceosomes in a two-step mechanism too. DHX35 could first translocate along the U2 snRNA, dissociating the U2/BS helix and priming the spliceosome for further remodeling. DHX15 then could act on the 3’-end of U6 snRNA to complete disassembly. Alternatively, or in addition, DHX38 (*sc*Prp16) could play a role in proofreading spliceosomes for quality control as suggested earlier:^42,71^ As a result of an inefficient docking of the U2 snRNA or the BS-A, DHX38 could revert a transient C/C* complex to a B* complex, which could further rearrange to B*^Q^ upon binding of DHX15–TFIP11–GCFC2. In this scenario, GPATCH1–DHX35 could already be present at the C/C* stage, as was suggested before. As a result of the concerted action of DHX35 (release of the pre-mRNA intron from the U2 snRNA) and DHX15 (release of U6 snRNA), the non-productive spliceosome could be recycled and the splicing factors reused for another round of splicing.

The presence of GPATCH1–DHX35 in catalytically active spliceosomes in human,^26^ *C. neoformans*^24^ and *S. pombe*,^21,23^ as well as their requirement for proper canonical splicing, indicates that they have a regulatory role in maintaining splicing fidelity. Alternative splicing was increased upon deletion of GPATCH1 or DHX35 in *C. neoformans*,^24^ as well as upon deletion of GPATCH1, DHX35 or WDR83 in *S. pombe*.^22,23^ The interaction surface between GPATCH1 and the spliceosome reported here, must differ from its binding site in C or C* spliceosomes due to several steric clashes. Here, GPATCH1 could tether DHX35 to the spliceosome periphery, possibly aided by WDR83. This is in line with a previous report from *S. pombe*, where GPATCH1 binds to DHX35 via its G-patch domain and to WDR83 via its C-terminus, while WDR83 contributed to the recruitment of DHX35 to spliceosomes.^23^ Does the role of GPATCH1–DHX35 within C* spliceosomes or the C to C* transition extend beyond proofreading? This is an intriguing question to be addressed in future studies.

While this manuscript was under preparation, structures of catalytically activated spliceosomes from *S. pombe* were reported (termed B^d^ complexes), which also represent aberrant spliceosomal complexes containing *sp*Glp1 (*ct*GPATCH1) and *sp*Gih35 (*ct*DHX35),^59^ with notable differences: *sp*Prp43 (*ct*DHX15) was not identified in the cryo-EM structures, possibly because *sp*Prp43’s binding platform, including *sp*Sde2 (*ct*SDE2), was not fully assembled. Soni et al. reported a one nucleotide insertion at the pre-mRNAs 5′ss, resulting in a 5′ss bulge at the active site, recognized by *sp*Glp1 in a similar manner as in our B*^Q^ complex. This suggests that errors in 5′ss positioning due to misalignment by the U5/5’-exon or U6/5′-intron duplex can lead to a 5′ss bulge and consequently GPATCH1 recruitment. However, as the target of *sp*Gih35 in the B^d^ complex could not be identified, the role of *sp*Gih35 in these complexes remained enigmatic. In our study, we show that DHX35 unwinds the U2/BS helix in aberrant spliceosomes, thus assigning a previously unobserved role for DHX35 helicase. Since the position of *sp*Gih35 in the B^d^ complex is almost superimposable with our structure, it is plausible to assume that GPATCH1 and DHX35 likely play a similar proofreading role across species by dissociating the U2/BS helix. This in turn suggests that the occurrence of an aberrant 5′ss bulge at the active site of spliceosomes could be a more general accuracy problem.

Consistent with previous observation,^32^ our structures suggest that the ILS and B*^Q^ complexes are primed for disassembly, but require ATP to initiate translocation by DHX35 and DHX15. However, other factors than the ones identified in our cryo-EM structures might be required for proper spliceosome disassembly and possible re-assembly. While we cannot rule out that the B*^Q^ complexes might be targeted for degradation of the aberrant splicing substrate, as suggested by Soni et al. in *S. pombe*,^59^ it is also possible, that rearrangements or a new round of assembly on the suboptimal pre-mRNA could lead to a productive splicing reaction, following the quality control mechanism proposed in our study. Future studies, including RNA sequencing of transcripts that are found in B*^Q^ spliceosomes, and more broadly in catalytically active spliceosomes associated with the GPATCH1–DHX35 complex, followed by analysis of their intron sequences, will provide deeper insights into the biological role of the B*^Q^ complexes.

In conclusion, our data have revealed that the two RNA helicase complexes DHX35–GPATCH1 and DHX15–TFIP11 are key players in the coordinated disintegration of rejected spliceosomes by linking 5′ss proofreading with spliceosome disassembly.

## Materials and methods

### Bacterial strains

For plasmid construction, the *Escherichia coli* Dh5α (ThermoFisher Scientific) strain was used.

### C. *thermophilum* strains and culturing

*C. thermophilum* strains P_ACT1_-ERG1-T_GPD_/P_ACT1_-ProtA-TEV-FLAG-DHX15-T_CTHT_0004700_, P_ACT1_-ERG1-T_GPD_/P_ACT1_-ProtA-TEV-FLAG-DHX35-T_CTHT_0004700_, P_ACT1_-ERG1-T_GPD_/P_ACT1_-ProtA-TEV-FLAG-WDR83-T_CTHT_0004700_, P_ACT1_-ERG1-T_GPD_/P_ACT1_-TFIP11-FLAG-TEV-ProtA-T_CTHT_0004700_ and P_ACT1_-ERG1-T_GPD_/P_ACT1_-GCFC2-FLAG-TEV-ProtA-T_CTHT_0004700_ used in this study were derived from the DSM 1495 wild-type strain (DSMZ, Braunschweig, Germany) and were generated and cultured as described previously.^72^

### Affinity purification of *C. thermophilum* spliceosomes

Mycelium from *C. thermophilum* strains expressing protA-TEV-FLAG-tagged baits was harvested, washed in water, dried under vacuum and frozen in liquid nitrogen. Frozen mycelium was mechanically disrupted using a cryogenic grinding mill (Retsch MM400) in lysis buffer (60 mM Tris-HCl, pH 8.0, 40 mM KCl, 50 mM NaCl, 2 mM MgCl_2_, 5% glycerol, 1 mM DTT, 0.1% NP-40, EDTA-free protease inhibitors (SIGMA*FAST*), 0.013 U/µL RiboLock RNase Inhibitors (ThermoFisher Scientific)). The lysate was centrifuged twice (10 min at 5000 rpm followed by 20 min at 17,000 rpm, 4 °C) and incubated with immunoglobulin G Sepharose 6 Fast Flow beads (GE Healthcare) for 12 h at 4 °C, followed by washing with 15 mL wash buffer (60 mM Tris-HCl, pH 8.0, 40 mM KCl, 50 mM NaCl, 2 mM MgCl_2_, 5% glycerol, 1 mM DTT, 0.01% NP-40). Bound proteins were eluted by TEV protease cleavage at 16 °C for 2 h in wash buffer supplemented with 1 U/µL RiboLock RNase Inhibitors (ThermoFisher Scientific). The TEV eluate was then loaded onto Flag-agarose beads (Anti-Flag M2 Affinity Gel, Sigma-Aldrich) and incubated for 12 h at 4 °C. Beads were washed with 10 mL wash buffer and eluted with buffer containing the Flag peptide. The elution buffer for cryo-EM analysis contained 60 mM Tris-HCl (pH 8.0), 40 mM KCl, 50 mM NaCl, 5 mM MgCl_2_, 2% glycerol, 0.01% NP-40, and 1 mM DTT.

### Mass spectrometry

The final FLAG eluate was separated on a 4%–12% polyacrylamide gel (NUPAGE, Invitrogen) and protein bands were visualized by colloidal Coomassie staining. FingerPrints proteomics (University of Dundee, UK) conducted semiquantitative Mass spectrometry. Co-precipitating proteins were identified with 1D nLC-ESI-MS-MS. MaxQuant software^73^ was used to analyze raw data.

### Assignment of the conserved splicing factor orthologs in *C. thermophilum*

We first surveyed The Spliceosome Database,^74^ The Alliance of Genomes Resources,^75^ The *Saccharomyces* Genome Database^76^ and PomBase^77^ to create a list of genes encoding spliceosomal proteins in human and baker’s yeast according to recently published catalogs of proteins involved in splicing.^26,57^ Then we assigned the *C. thermophilum* ortholog genes by using OrthoDB.^78^ U snRNA sequences were retrieved from RNAcentral.^79^

### Genome editing for dTAG endogenous knock-in

The procedures for generating the dTAG endogenous knock-in cell line were performed according to previously established criteria.^50^ Briefly, mouse embryonic stem cells (mESCs) were used for this purpose. Cells were seeded in a six-well plate the day before transfection to ensure they were in exponential growth on the day of transfection. On the next day, cells were transfected with PITCh plasmids using Lipofectamine 3000 (Invitrogen) following the manufacturer’s protocol. The PITCh plasmids included the following: pX459-sgRNA (Addgene: #62988) for targeting specific genomic sites within target genes (*DHX35* and *GPATCH1*), pUC57-DHX35/GPATCH1-dTAG donor containing the dTAG microhomology repair template, and pX459-sgPITCh (Addgene: #62988) for cutting and fetching out the repair template. The used gRNA sequences are: *DHX35*, AGTGAGTGGAGCTCGGCAAG; *GPATCH1*, GCTGGACAGTGACAGCGACG. After transfection, cells were diluted and cultured in the presence of 1–2 μg/mL puromycin (Meilunbio) for 10–14 days in 10-cm plates. Single surviving colonies were manually picked into 96-well plates, expanded, and subjected to genotype confirmation using PCR analysis and western blotting assay. The effectiveness of protein degradation in positive clones was validated through dTAG treatment, followed by western blotting analysis. At least three clones were selected and retained for further experiments. Anti-Flag (Proteintech, #66008-4-Ig) and anti-Tubulin (Proteintech, #11224-1-AP) antibodies were used in this study.

### RNA-sequencing library preparation

RNA-sequencing libraries were prepared using DHX35-dTAG or GPATCH1-dTAG mESC cell lines. Cells were treated with either DMSO (control) or dTAG-13 (Tocris) at a final concentration of 200 nM for 4 h to induce protein degradation. Total RNA was extracted using TRIzol reagent (Invitrogen) according to the manufacturer’s protocol. Human *DLD-1* total RNA was included as a spike-in control and mixed with the extracted RNA samples for normalization during downstream sequencing analysis. Ribosomal RNA (rRNA) was removed using an rRNA depletion kit as described previously.^80^ The remaining RNA was used for subsequent sequencing libraries preparation with Stranded mRNA-seq Lib Prep Module for Illumina (Abclonal). Sequencing was performed on DNBSEQ-T7 platform with PE150 strategy (GenePlus Technology, China).

### RNA sequencing analysis

The paired raw reads were quality-checked using FastQC (v0.11.9) (Babraham Institute). Adapter sequences and low-quality reads were trimmed using Trim Galore (v0.6.6) (Babraham Institute) with the parameter -q 25. The trimmed reads were then aligned to the mouse mm10 and human hg19 assemblies using STAR (v2.7.5c),^81^ following the preprocessing instructions specified for JUM.^82^

Differentially spliced introns were identified using JUM (v3.0.0),^82^ with splicing events deemed significant at a *P*-value < 0.05. Additionally, a minimum threshold of 5 supporting reads was applied to ensure event reliability. Consensus sequence logos were generated using the R package ggseqLogo (v0.2).^83^

### Electron microscopy and image processing

A 3.5 µL sample of purified DHX15 sample was applied to Quantifoil R1.2/1.3 holey-carbon grids (pre-coated with 2 nm carbon). The grids were blotted for 4–5 s at 4 °C and plunge-frozen in liquid ethane using a FEI Vitrobot Mark IV. Data collection was performed on a Titan Krios G4 cryo-electron microscope operating at 300 keV, using EPU 2. Micrographs were recorded with a pixel size of 0.932 Å and a defocus range of –1 to –2.5 µm, using a Falcon IV direct electron detector in EER format under low-dose conditions (total dose ~50 e^–^/Å^2^). Original image stacks were dose-weighted, aligned, summed, and drift-corrected using MotionCor2.^84^ Contrast-transfer function (CTF) parameters and resolutions were estimated for each micrograph using CTFFIND4 and GCTF, respectively.^85,86^ Micrographs with an estimated resolution of less than 5 Å and an astigmatism of less than 5% were manually screened for contamination or carbon rupture.

A total of 34,287 good micrographs were selected. Particle picking was performed automatically using Gautomatch v0.56 without using a reference, yielding 3,766,153 particles. The picked particles were extracted in Relion v5.0^87^ (rescaled to a pixel size of 2.796 Å) and subsequently imported into cryoSPARC v4.5.3. To facilitate computing, the particles were divided into three subsets for heterogeneous refinement using the baker’s yeast ILS complex as a reference (PDB: 5Y88).^33^ After refinement, 741,073 particles displaying clear high-resolution features were selected and re-imported into Relion v5.0 for 3D classification (three rounds in total). During the first round of alignment-free 3D classification, particles were sorted into four classes using a 400 Å diameter spherical mask automatically generated in Relion v5.0 (T-value = 4). Three classes displaying clear ILS features or DHX35 density were selected for further refinement. A second round of focused 3D classification was performed using a focused spherical mask (*d* = 80 Å) around DHX35 (T-value = 20). Class 2 was divided into three sub-classes, while classes 3 and 4 were divided into four sub-classes each. In the end, 16,442 particles from class 2 were identified as state B*^Q2^, 77,668 particles represented a high-resolution ILS state, and 66,478 particles from class 4 were designated as state B*^Q1^. For additional sorting (third round) of the DHX15 region in state B*^Q2^, focused classification with a spherical mask around DHX15 was applied, resulting in four classes. One class with pronounced DHX15 density was designated as the B*^Q2^ (focus DHX15). Notably, this class was used exclusively to visualize DHX15, thus no molecular model was provided. Similarly, focused classification using sphere mask around TFIP11 was applied in state B*^Q1^ to enhance the density map of TFIP11. The resulting map was only used to verify the rigid-body fit of the TFIP11 model. The detailed sorting scheme is illustrated in Supplementary information, Fig. S1.

In the end, the selected classes (ILS, B*^Q1^ and B*^Q2^) were re-extracted in Relion (pixel size 0.932 Å) and refined to final states in Relion v5.0.^87^ To obtain the final reconstruction, CTF refinement and multibody refinement were used. For state ILS, the map was segmented into four parts: the U5 snRNP region, the spliceosome body region, the AQR and U2 snRNP region, and the SYF1 region. For state B*Q1, the map was divided into three parts: the spliceosome body region, the IBC region, and the DHX35 region. For state B*^Q2^, the map was segmented into two parts: the spliceosome body region and the DHX35 region. The final maps were post-processed and local-resolution-filtered using masks that were automatically generated in Relion v5.0.^87^ The final maps were also sharpened using DeepEMhancer v0.13.^88^

### Model building and refinement

To generate the molecular model of the *C. thermophilum* spliceosome ILS complex, the structures of baker’s yeast and human ILS spliceosomes (PDB IDs: 5Y88 and 6ID1)^33,89^ were used as initial references for positioning splicing factors. The predicted structures of *C. thermophilum* splicing factors were obtained from the AlphaFold database.^47^ Initially, the baker’s yeast and human ILS complexes were fitted as rigid bodies into the cryo-EM density map in Coot v0.9.5.^90^ Subsequently, the *C. thermophilum* splicing factors were aligned to the baker’s yeast or human models of the ILS complex. All splicing factors were then manually assembled to form the preliminary model.

To get the final model for the *ct*ILS complex, manual adjustments were performed in Coot v0.9.5,^90^ using the post-processed density maps from the Relion v5.0 multibody refinement. For regions with sufficient resolution to visualize side chains, AlphaFold models were adjusted to fit the density. In peripheral regions that show lower resolution, AlphaFold models were retained but had their side chains removed (e.g., U2 snRNP, AQR, PRP19 complex). For regions lacking corresponding density, those regions were completely excluded from the final model. The TFIP11–GCTC2 and DHX15–TFIP11 complexes were modeled using AlphaFold3 server^91^ and fitted into the density maps through rigid-body fit. Sequences for U2, U5, and U6 snRNAs were downloaded from RNAcentral.^79^ The human models for U2, U5, and U6 snRNAs were manually mutated to match the *C. thermophilum* sequences and adjusted to fit the density. Since the sample was purified from in vivo sources, the pre-mRNA in the structure represented a mixture of substrates, and thus, besides the well conserved splicing motif, the remaining sequence was modeled as unknown bases.

For the *ct*B*^Q1^ and *ct*B*^Q2^ states, the final *C. thermophilum* ILS model was used as the initial reference. Proteins absent from the ILS complex, such as CWC22 and SRRM2, were modeled using AlphaFold predictions and fitted via rigid-body fit, followed by manual adjustments in Coot v0.9.5.^90^ The DHX35–GPATCH1 complex was also predicted by AlphaFold3 server^91^ and adjusted manually to fit into density map, with non-interacting regions removed. The remaining GPATCH1 structure, different conformations of U2 snRNA and the suboptimal pre-mRNA in the active center were manually built based on the density maps. In state B*^Q2^, due to low local resolution around the 5’ss/U6 helix, the model from the ILS complex was retained without further adjustments. Additionally, the CWF19, which is only present in the ILS complex, was removed for these states. Due to the low local resolution, the 3′-end of the U2 snRNP was excluded from the final model. Additionally, the TFIP11–GCFC2 complex in these two states was modeled as unknown residues.

The final models were real-space refined with secondary structure restraints using the PHENIX suite v1.19.^92^ Final model evaluation was performed with MolProbity.^93^ Maps and models were visualized and figures were created with ChimeraX v1.8.^94^

## Supplementary information

Supplementary information, Figure S1

Supplementary information, Figure S2

Supplementary information, Figure S3

Supplementary information, Figure S4

Supplementary information, Figure S5

Supplementary information, Figure S6

Supplementary information, Figure S7

Supplementary information, Figure S8

Supplementary information, Figure S9

Supplementary information, Figure S10

Supplementary information, Figure S11

Supplementary information, Figure S12

Supplementary information, Figure S13

Supplementary information, Figure S14

Supplementary information, Tables S1

Supplementary information, Tables S2

Supplementary information, Tables S3

Supplementary information, Tables S4

Supplementary figure legend

## Data Availability

All cryo-EM maps and molecular models were deposited in the Electron Microscopy Data Bank (EMDB) and in the Protein Data Bank (PDB) with accession codes: EMD-62841 and 9L5R for state ILS; EMD-62842 and 9L5S for state B*^Q1^; EMD-62843 and 9L5T for state B*^Q2^; EMD-62844 for state B*^Q2^ (focus DHX15). The RNA sequencing raw data have been deposited at GEO with accession code: GSE287473.
