# Supplementary material for: Structural insights into spliceosome fidelity: DHX35–GPATCH1- mediated rejection of aberrant splicing substrates
Source: Cell Res. 2025 Feb 28;35(4):296–308. doi: 10.1038/s41422-025-01084-w (PMC11958768; doi:10.1038/s41422-025-01084-w)
Supplement: Supplementary file 1 — Supplementary information, Figure S1 [file 41422_2025_1084_MOESM1_ESM.pdf]

a

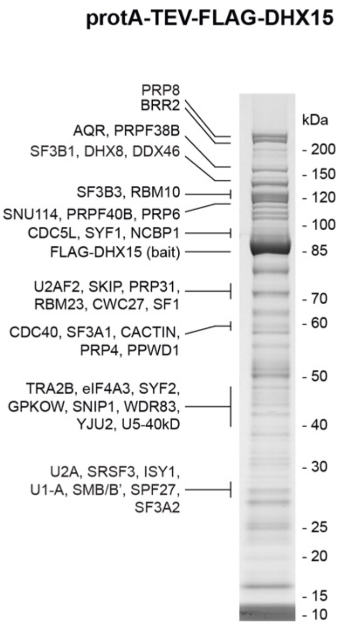

b Dataset: DHX15 WT

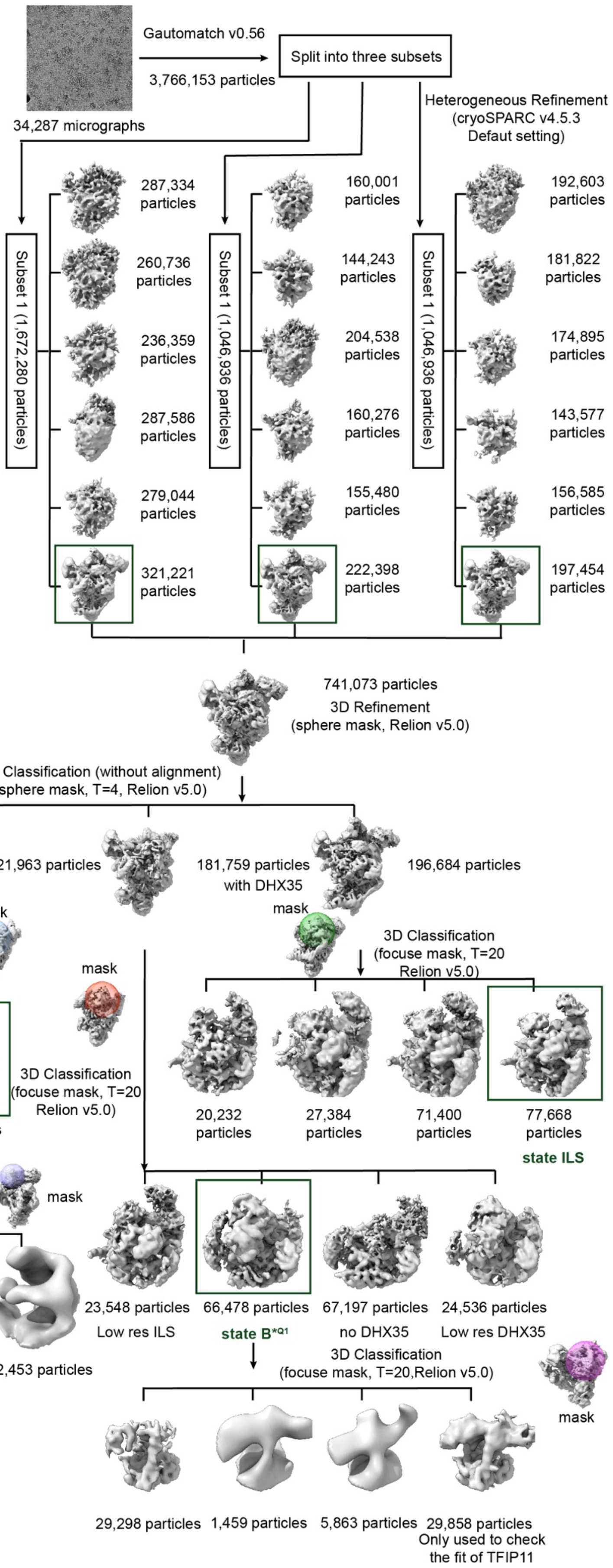

Figure S1: Cryo-EM data processing of the DHX15 sample.

a, Tandem-affinity purification of Protein A-TEV-FLAG-*ct*DHX15 from *C. thermophilum* lysates. The final FLAG-eluate was analyzed by SDS-PAGE and Coomassie-staining. Spliceosomal proteins were identified by mass spectrometry and labeled on the left according to their predicted molecular weights. The molecular weights (in kDa) of a protein standard are indicated on the right. **b**, Sorting scheme summarizing the data processing workflow for the DHX15 dataset. The masks, software and the key parameters used during various steps of data processing are indicated, alongside the schematic illustrations. Four classes were selected for final refinement and defined as B^{*Q1}, B^{*Q2}, B^{*Q2} (focus DHX15) and ILS complexes. The state B^{*Q2} (focus DHX15) map, and the map generated after focused classification of TFIP11 in state B^{*Q1}, were used to verify the rigid-body fit of DHX15 and TFIP11, respectively.
