# Supplementary material for: Structural insights into spliceosome fidelity: DHX35–GPATCH1- mediated rejection of aberrant splicing substrates
Source: Cell Res. 2025 Feb 28;35(4):296–308. doi: 10.1038/s41422-025-01084-w (PMC11958768; doi:10.1038/s41422-025-01084-w)
Supplement: Supplementary file 2 — Supplementary information, Figure S2 [file 41422_2025_1084_MOESM2_ESM.pdf]

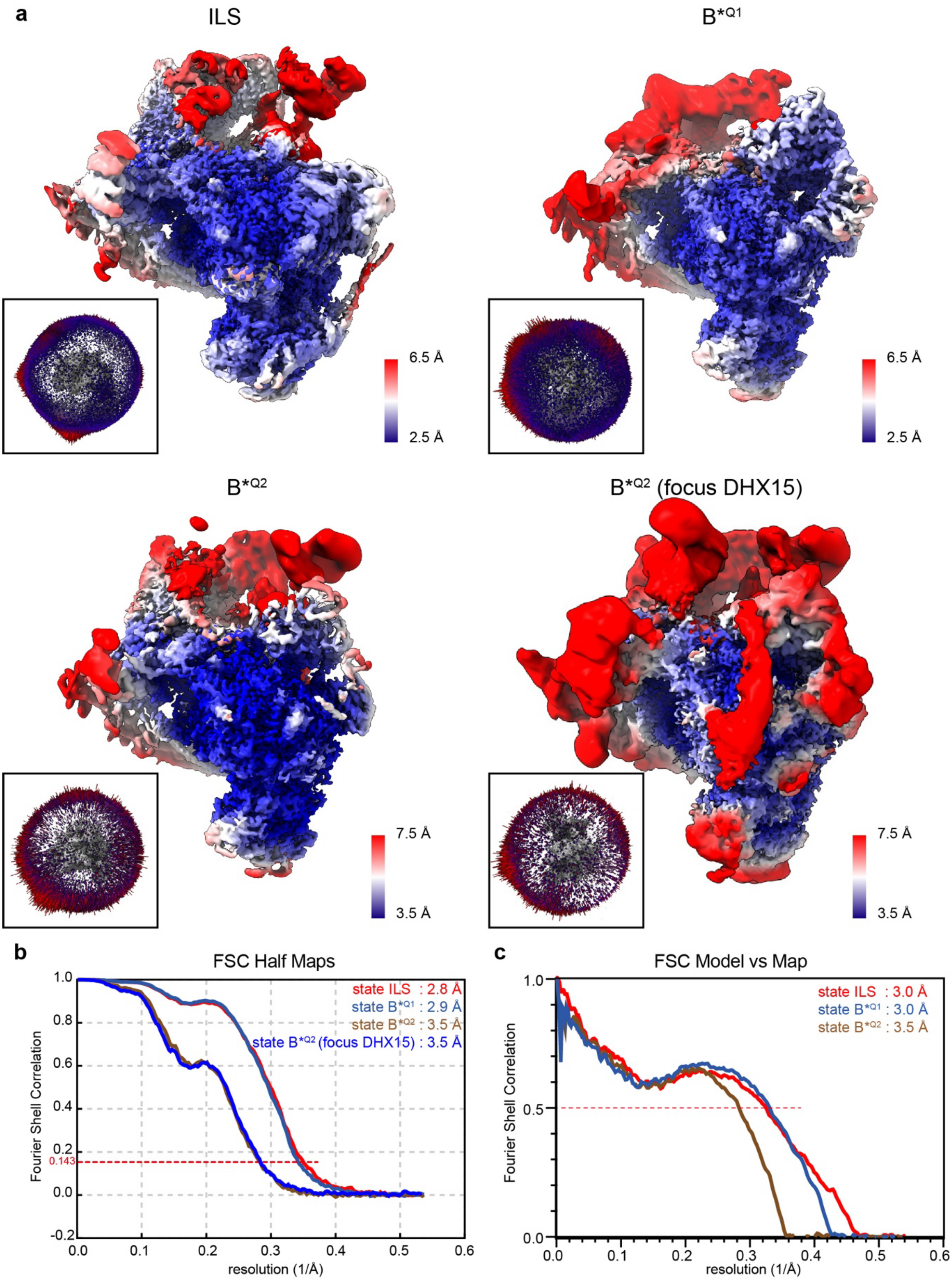

Figure S2: Cryo-EM structural analysis of DHX15 sample.

a, The local-resolution distributions and angle distributions of the four maps, as estimated by Relion and indicated by a blue-to-red color scale. **d**, Fourier shell correlation (FSC) curves of half-maps for ILS, B^{*Q1}, B^{*Q2} and B^{*Q2} (focus DHX15)

complexes. The FSC cutoff criterion of 0.143 was used for corresponding resolution estimation. **e**, FSC curves comparing the model to the map for ILS, B^{*Q1} and B^{*Q2} complexes.
