# Supplementary material for: Structural insights into spliceosome fidelity: DHX35–GPATCH1- mediated rejection of aberrant splicing substrates
Source: Cell Res. 2025 Feb 28;35(4):296–308. doi: 10.1038/s41422-025-01084-w (PMC11958768; doi:10.1038/s41422-025-01084-w)
Supplement: Supplementary file 3 — Supplementary information, Figure S3 [file 41422_2025_1084_MOESM3_ESM.pdf]

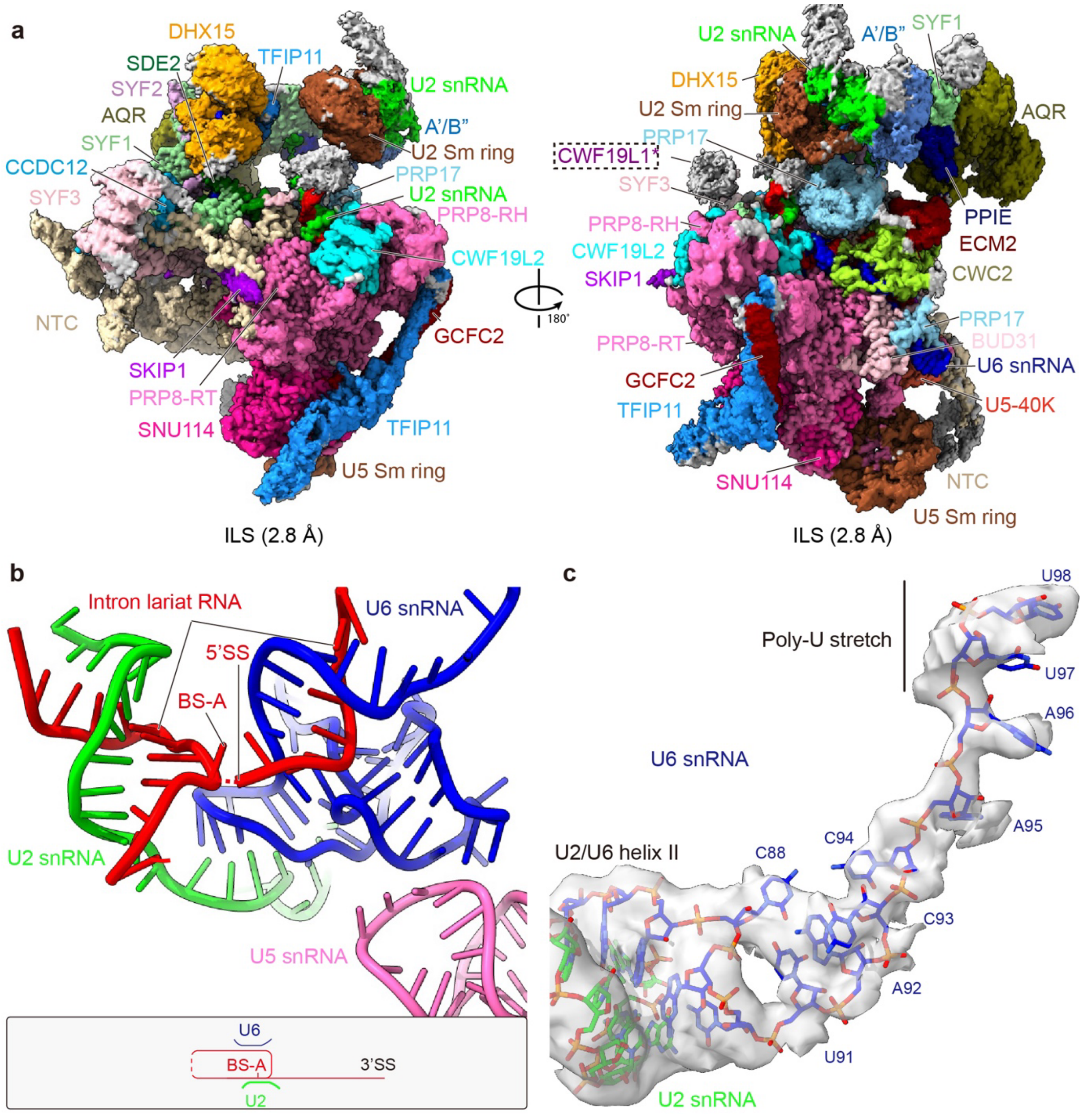

Figure S3: Structural details of the *ct*ILS state.

a, Two different views of the cryo-EM density maps of the ILS complex of *C. thermophilum* observed after affinity purification of DHX15. CWF19L1 is boxed and marked with an asterisk to indicate that its position cannot be assigned unambiguously. **b**, Models of the U2 (green), U5 (pink) and U6 (blue) snRNA with the intron-lariat RNA. A simplified scheme of the RNA interactions is illustrated in the bottom row. **c**, The resolution of the U2 and U6 snRNA, illustrated with the density maps, allows the identification of the bases in the 3' end of the U6 snRNA. The U2/U6 helix II and the poly-U stretch are indicated.
