# Supplementary material for: Structural insights into spliceosome fidelity: DHX35–GPATCH1- mediated rejection of aberrant splicing substrates
Source: Cell Res. 2025 Feb 28;35(4):296–308. doi: 10.1038/s41422-025-01084-w (PMC11958768; doi:10.1038/s41422-025-01084-w)
Supplement: Supplementary file 4 — Supplementary information, Figure S4 [file 41422_2025_1084_MOESM4_ESM.pdf]

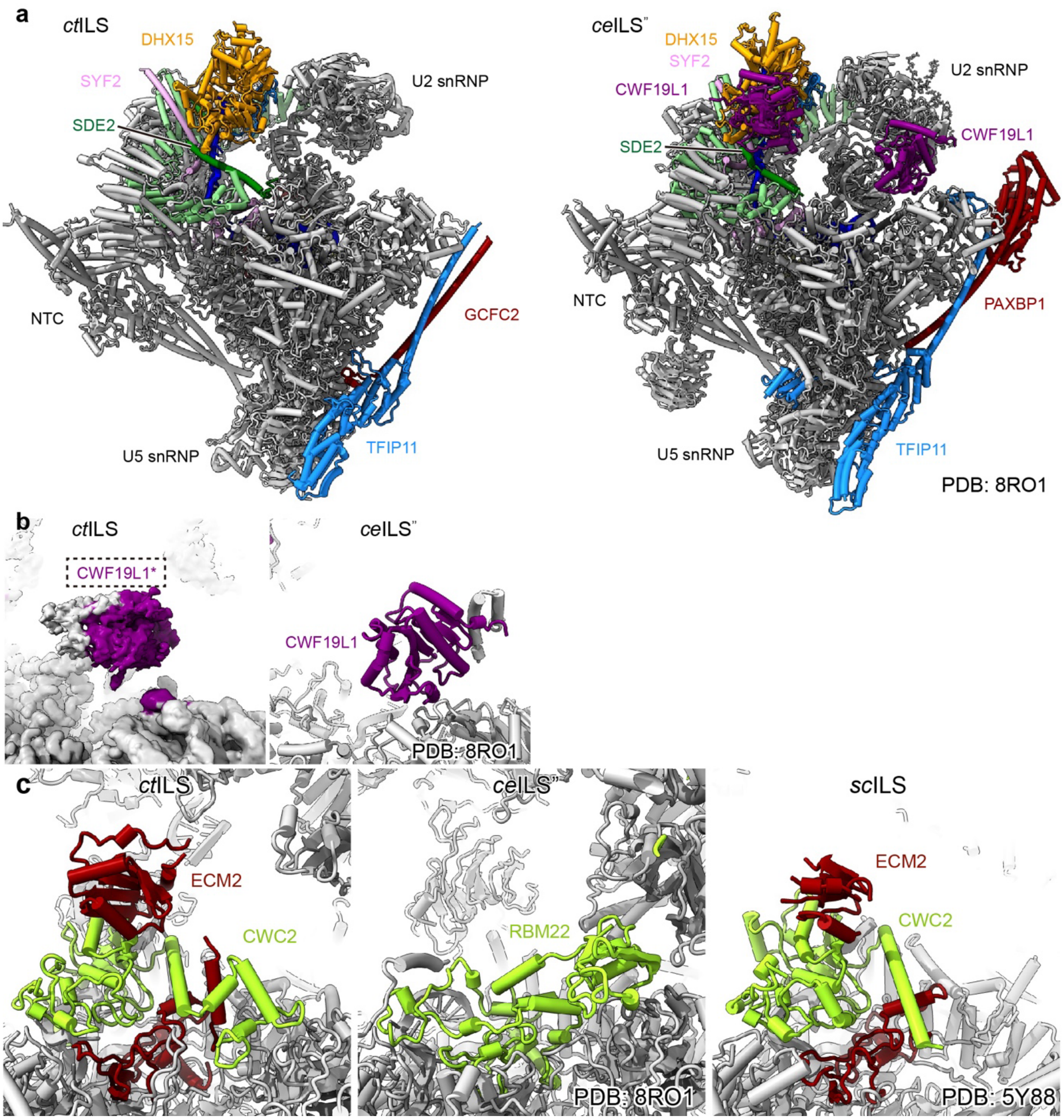

Figure S4: Structural comparison of the *ct*ILS complex with *ce*ILS".

a, Structure comparison between the *ct*ILS (left) and *ce*ILS" complexes (right, *C. elegans* ILS complex, PDB: 8I0R). Highlighted components: GCFC2 (red), TFIP11 (light blue), DHX15 (orange), SYF2 (pink), SDE2 (light green), PAXBP1 (red), CWF19L (purple), and PPIE (blue). *ct*ILS lacks CWF19L1 and PPIE, which are present in the *ce*ILS" complex. **b,** Close-up views of key regions in *ct*ILS (left) and *ce*ILS" (right) with focus on CWF19L1 (purple). In the ctILS state, CWF19L1 is boxed and marked with an asterisk to indicate that its position cannot be assigned unambiguously. **c**, Close-up views of key regions in *ct*ILS (left), *ce*ILS" (midddle) and scILS (right) with focus on RBM22 in *ce*ILS and ECM2/CWC2 in *ct* and *sc* ILS complexes.
