# Supplementary material for: Structural insights into spliceosome fidelity: DHX35–GPATCH1- mediated rejection of aberrant splicing substrates
Source: Cell Res. 2025 Feb 28;35(4):296–308. doi: 10.1038/s41422-025-01084-w (PMC11958768; doi:10.1038/s41422-025-01084-w)
Supplement: Supplementary file 5 — Supplementary information, Figure S5 [file 41422_2025_1084_MOESM5_ESM.pdf]

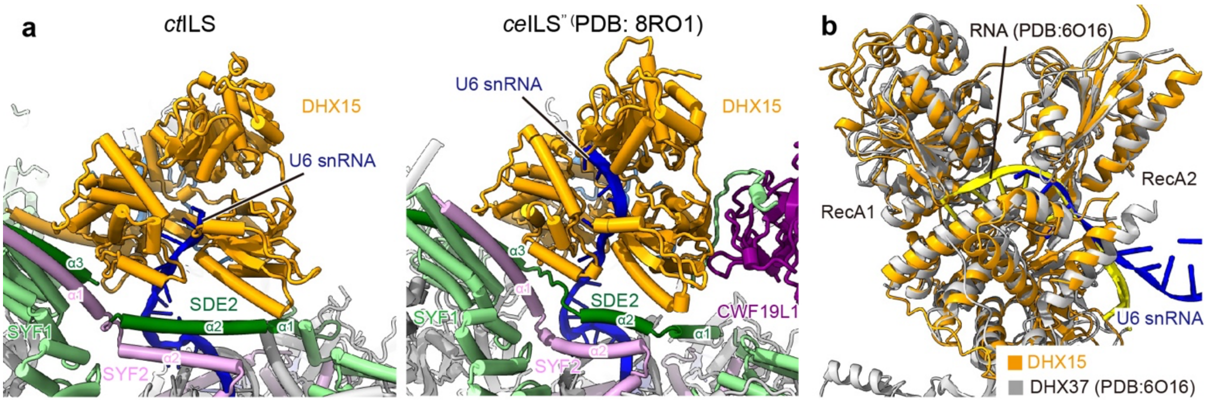

Figure S5: Structural comparison of the DHX15 binding region in the *ct*ILS and *ce*ILS" complex.

a, Comparison between binding interface of DHX15 in the *ct*ILS (left) and *ce*ILS" complexes (right, *C. elegans* ILS complex, PDB: 8I0R). Highlighted components: DHX15 (orange), SYF2 (pink), SDE2 (green), SYF1 (light green) and CWF19L (purple). The U6 snRNA is shown in blue. **b**, Superimposition of DHX15 (orange) with the crystal structure of nucleotide-free DHX37 (gray) bound to RNA (yellow, PDB: 6O16). U6 snRNA is shown in blue.
