# Supplementary material for: Structural insights into spliceosome fidelity: DHX35–GPATCH1- mediated rejection of aberrant splicing substrates
Source: Cell Res. 2025 Feb 28;35(4):296–308. doi: 10.1038/s41422-025-01084-w (PMC11958768; doi:10.1038/s41422-025-01084-w)
Supplement: Supplementary file 6 — Supplementary information, Figure S6 [file 41422_2025_1084_MOESM6_ESM.pdf]

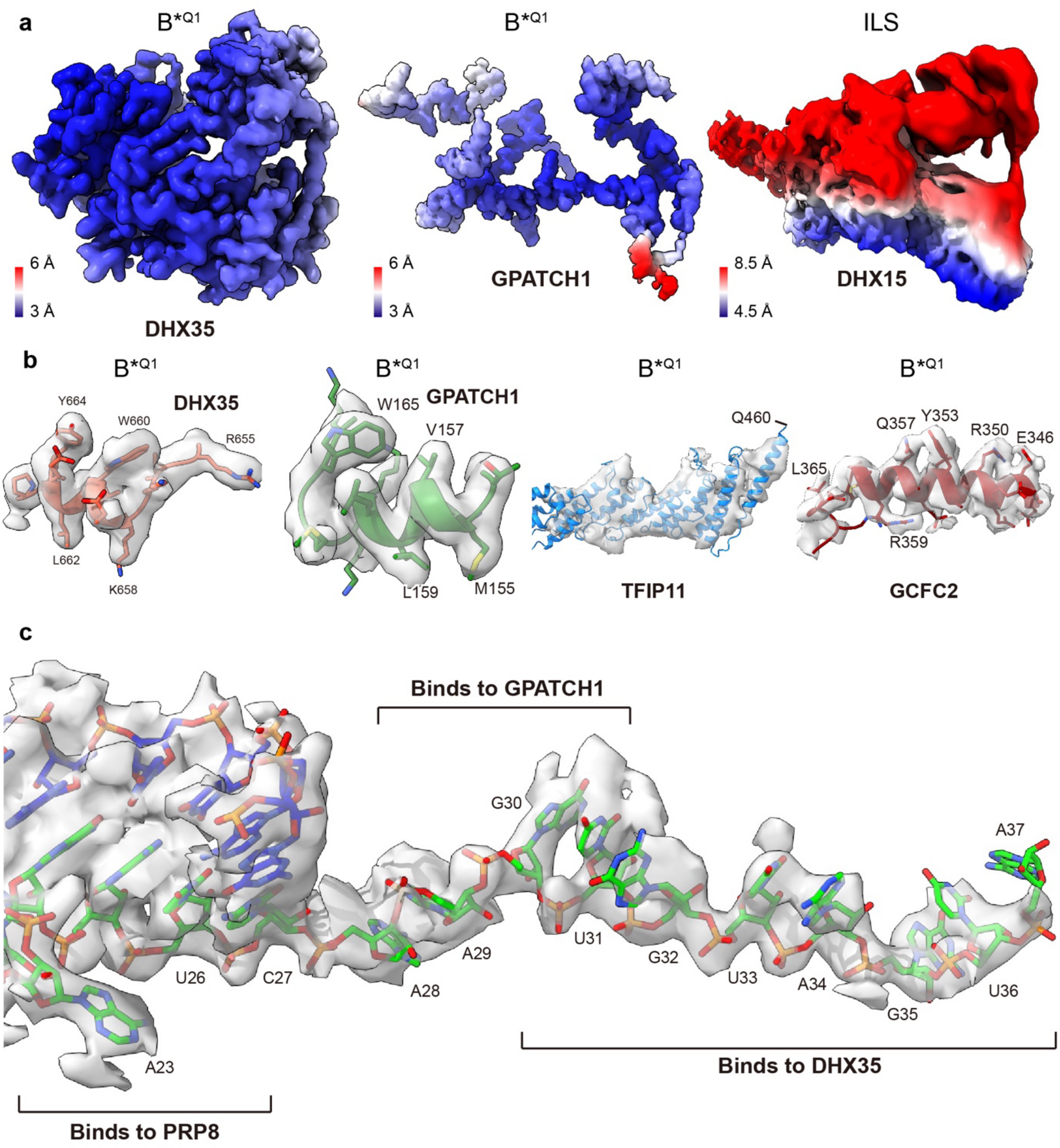

Figure S6: Structural details of DHX35, GPATCH1, TFIP11, GCFC2, DHX15 and U2 snRNA from the B*^{Q1} complex.

a, Local resolution maps of DHX35 (left) and GPATCH1 (middle) in the B*^{Q1} complex, and DHX15 (right) in the *ct*ILS state. The color bars indicate the local resolution ranges for each protein as estimated by Relion. **b**, Close-up views of the cryo-EM densities for DHX35 (left, aa. 655-666), GPATCH1 (middle left, aa. 155-166), and GCFC2 (right, aa. 345-367), highlighting that the resolution is sufficient to visualize all side chain details. Models are shown as sticks surrounded by transparent density maps. In the middle right, the overall view of the density map generated after focused classification

of TFIP11 in state B^{*Q1} shows the fit of TFIP11 into the density. **c**, The U2 and U6 snRNA are shown with the density maps. Interactions with PRP8, GPATCH1 and DHX35 are indicated.
