# Supplementary material for: Structural insights into spliceosome fidelity: DHX35–GPATCH1- mediated rejection of aberrant splicing substrates
Source: Cell Res. 2025 Feb 28;35(4):296–308. doi: 10.1038/s41422-025-01084-w (PMC11958768; doi:10.1038/s41422-025-01084-w)
Supplement: Supplementary file 7 — Supplementary information, Figure S7 [file 41422_2025_1084_MOESM7_ESM.pdf]

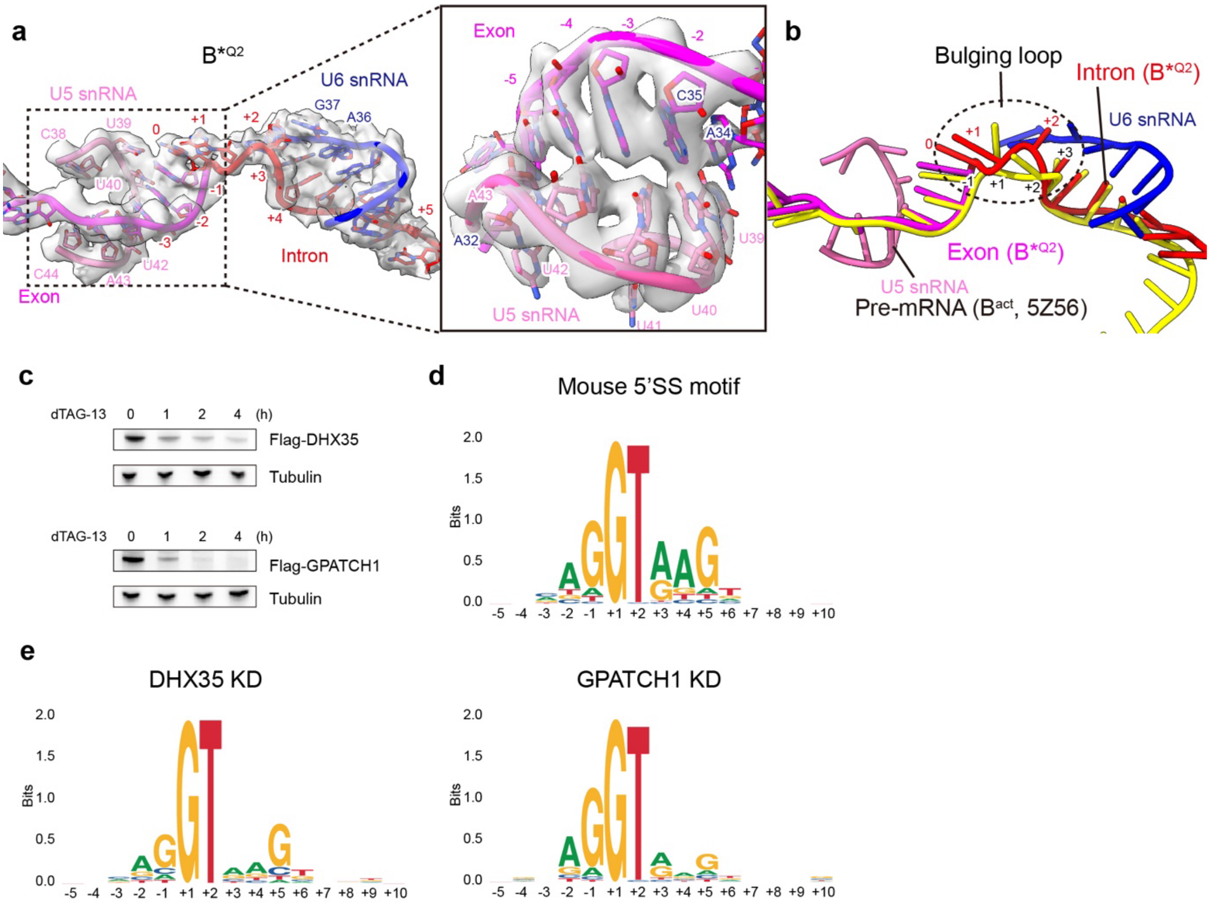

Figure S7: Structural and functional insights into the spliceosomal 5′ splice site.

a, Density map of the active site center RNA in *ct*B^{*Q2} with the nucleotide positions of the modelled RNAs indicated. Detailed interaction between the U5 snRNA and 5' exon is shown with density map as an insert. **b**, Comparison of *ct*B^{*Q2} pre-mRNA with a pre-mRNA molecule from the B^{act} complex (PDB: 5Z56) illustrating the bulging loop. **c**, Functional validation of DHX35 and GPATCH1. Western blot analysis of endogenous Flag-FKBP12^{F36V} tagged DHX35 and GPATCH1 protein levels over time (0–4 hours) after dTAG-13 treatment demonstrates effective protein degradation (top left panel) in mouse ESCs. The DHX35 and GPATCH1 proteins were detected using Anti-Flag antibody. Anti-tubulin antibody was used to assess equal loading. **d-e**, Sequence logos depict the 5′SS motifs derived from RNA sequencing of mouse ESCs from wt (**d**) and DHX35 and GPATCH1 depleted cells (**e**). Comparisons reveal alterations in 5'ss sequence preferences, particularly from positions +3 to +6, highlighting the regulatory roles of DHX35 and GPATCH1 in splicing fidelity.
