# Supplementary material for: Structural insights into spliceosome fidelity: DHX35–GPATCH1- mediated rejection of aberrant splicing substrates
Source: Cell Res. 2025 Feb 28;35(4):296–308. doi: 10.1038/s41422-025-01084-w (PMC11958768; doi:10.1038/s41422-025-01084-w)
Supplement: Supplementary file 8 — Supplementary information, Figure S8 [file 41422_2025_1084_MOESM8_ESM.pdf]

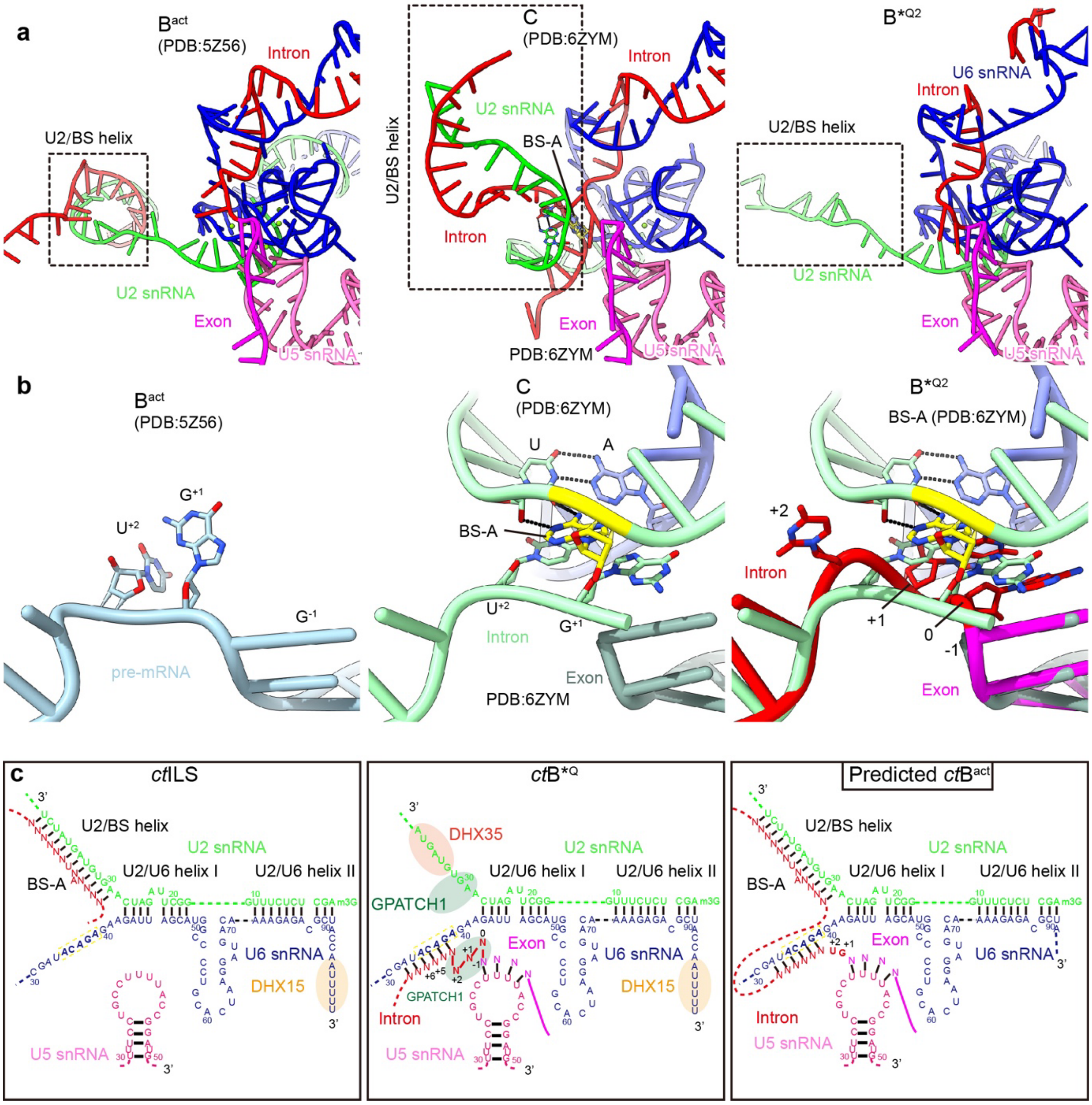

Figure S8: Suboptimal pre-mRNA in *ct*B^{*Q2} adopts an altered conformation in the active site center.

a, Close-up views of the splicing active center in *ct*B^{*Q2}, compared with two published states, *hs*B^{act} (PDB: 5Z56) and *hs*C (PDB: 6ZYM), showing different conformations of U2 and U6 snRNA. The U2 and BS helix undergoes a large conformational change to position the branch site adenosine (BS-A) in the active center in the B* complex (left to middle panel). The BS helix is released in the *ct*B^{*Q2} (right panel). **b**, Detailed view of the pre-mRNA in the splicing active center in *hs*B^{act} (PDB:5Z56) (left), *hs*C (PDB:6ZYM) (middle) and *ct*B^{*Q2} (right) complexes. The conformation of the pre-mRNA in *ct*B^{*Q2} spatially clashes with the BS-adenosine, placing *ct*B^{*Q2} in an inactive conformation. **c**, Schematic illustration of the RNA conformations within the active

center in the predicted *ct*B^{act}, *ct*B^{*Q} and *ct*ILS active site centres. Due to missing information on the pre-mRNA, its sequence is represented as poly “N.” The predicted RNA conformation in *ct*B^{act} is modeled based on the *hs*B^{act} structure (PDB: 5Z56).
