# Supplementary material for: Structural insights into spliceosome fidelity: DHX35–GPATCH1- mediated rejection of aberrant splicing substrates
Source: Cell Res. 2025 Feb 28;35(4):296–308. doi: 10.1038/s41422-025-01084-w (PMC11958768; doi:10.1038/s41422-025-01084-w)
Supplement: Supplementary file 9 — Supplementary information, Figure S9 [file 41422_2025_1084_MOESM9_ESM.pdf]

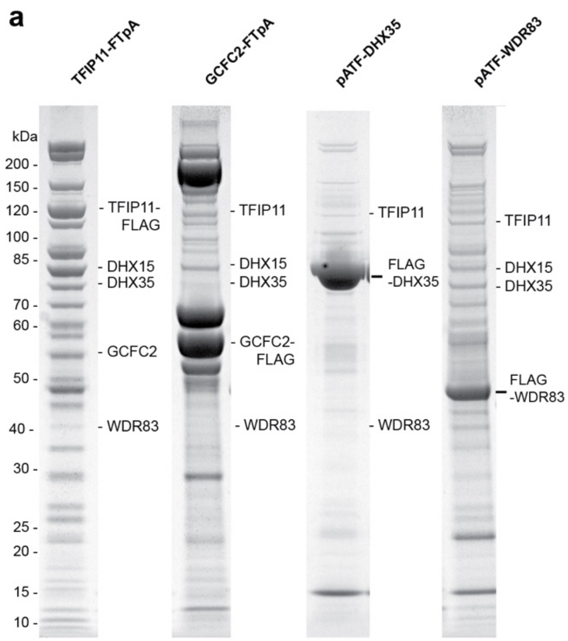

Figure S9: Purification of protA-TEV-FLAG-tagged spliceosomal components TFIP11, GCFC2, DHX35, and WDR83.

a, SDS-PAGE analysis of the co-purified proteins using protA-TEV-FLAG-tagged TFIP11, GCFC2, DHX35, and WDR83 proteins. FLAG-tagged TFIP11, GCFC2, DHX35, and WDR83 were expressed and purified from *C. thermophilum* using a similar approach as DHX15. The bait proteins are labeled. The purified samples were further analyzed by Mass spectrometry to identify co-purified proteins.
