# Supplementary material for: Structural insights into spliceosome fidelity: DHX35–GPATCH1- mediated rejection of aberrant splicing substrates
Source: Cell Res. 2025 Feb 28;35(4):296–308. doi: 10.1038/s41422-025-01084-w (PMC11958768; doi:10.1038/s41422-025-01084-w)
Supplement: Supplementary file 10 — Supplementary information, Figure S10 [file 41422_2025_1084_MOESM10_ESM.pdf]

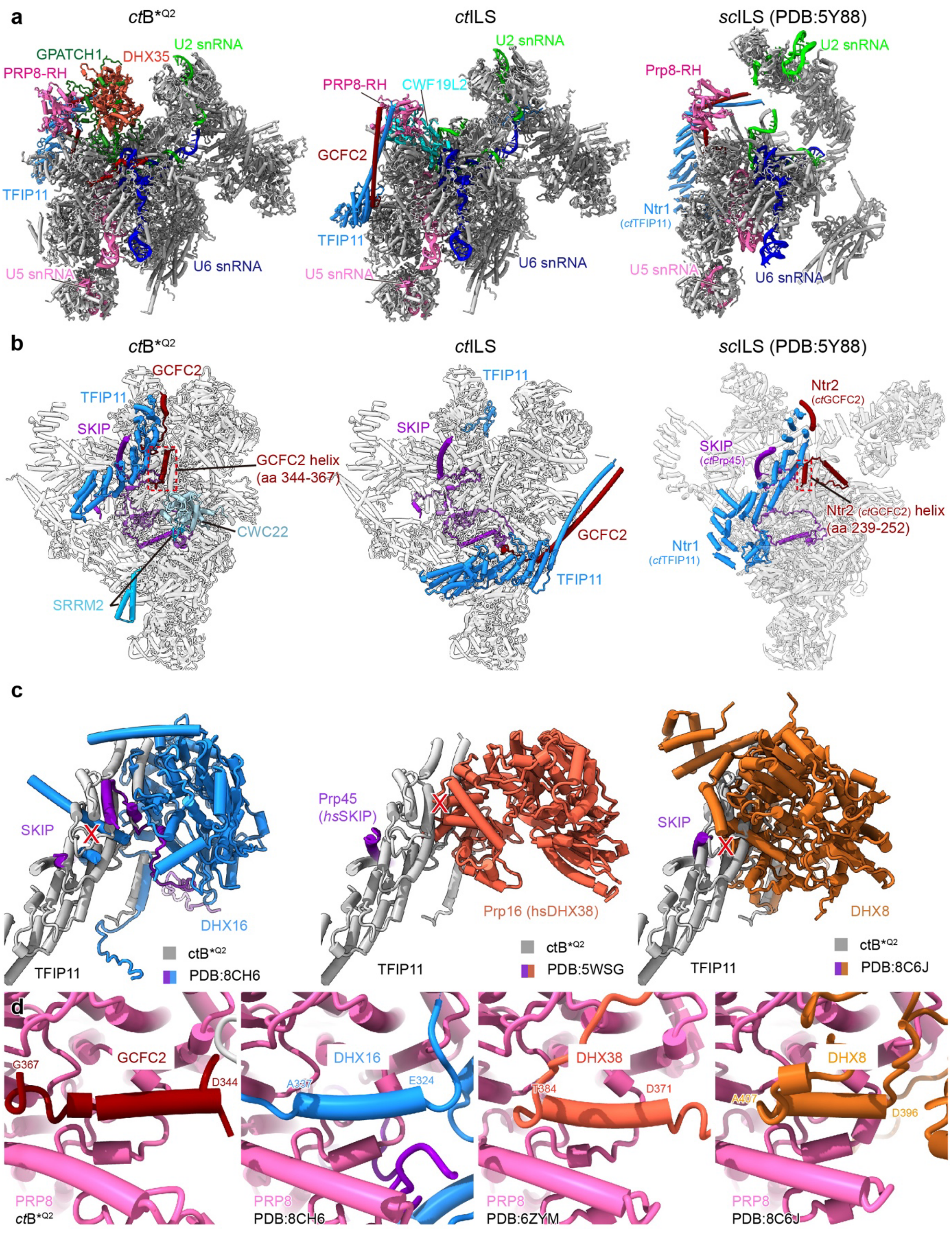

Figure S10: Structural comparison of the *ct*B*Q2, *ct*ILS and *sc*ILS complexes.

a, Differences between *ct*B*Q2 (left), *ct*ILS (middle) and *sc*ILS (right) are highlighted: PRP8-RH domain (hot pink), GPATCH1 (green), DHX35 (tomato red), and CWF19 (green). **b**, Comparison of the position of the TFIP11-GCFC2 dimer in the *ct*B*Q2 (left), *ct*ILS (middle) and *sc*ILS (right) complexes. SRRM2 and CWC22 are present in *ct*B*Q2

(left) but missing in *ct*ILS (right). Notably, TFIP11 binds to a similar region in *ct*B^{*Q2} as it does in baker's yeast *sc*ILS state. **c**, Overlay of TFIP11 from *ct*B^{*Q2} with the position of DHX16 (left, PDB: 8CH6), DHX38 (middle, PDB: 5WSG) and DHX8 (right, PDB: 8C6J) during B^{act} and C* complexes. **d**, Close-up view of the steric clash between GCFC2 (left, aa 344-367) and the binding sites for DHX16 (aa 324-337, PDB: 8CH6), DHX38 (aa 371-384, PDB: 6ZYM) and DHX8 (aa 396-407, PDB: 8C6J).
