# Supplementary figures and images for: Structural insights into spliceosome fidelity: DHX35–GPATCH1- mediated rejection of aberrant splicing substrates
Source: Cell Res. 2025 Feb 28;35(4):296–308. doi: 10.1038/s41422-025-01084-w (PMC11958768; doi:10.1038/s41422-025-01084-w)

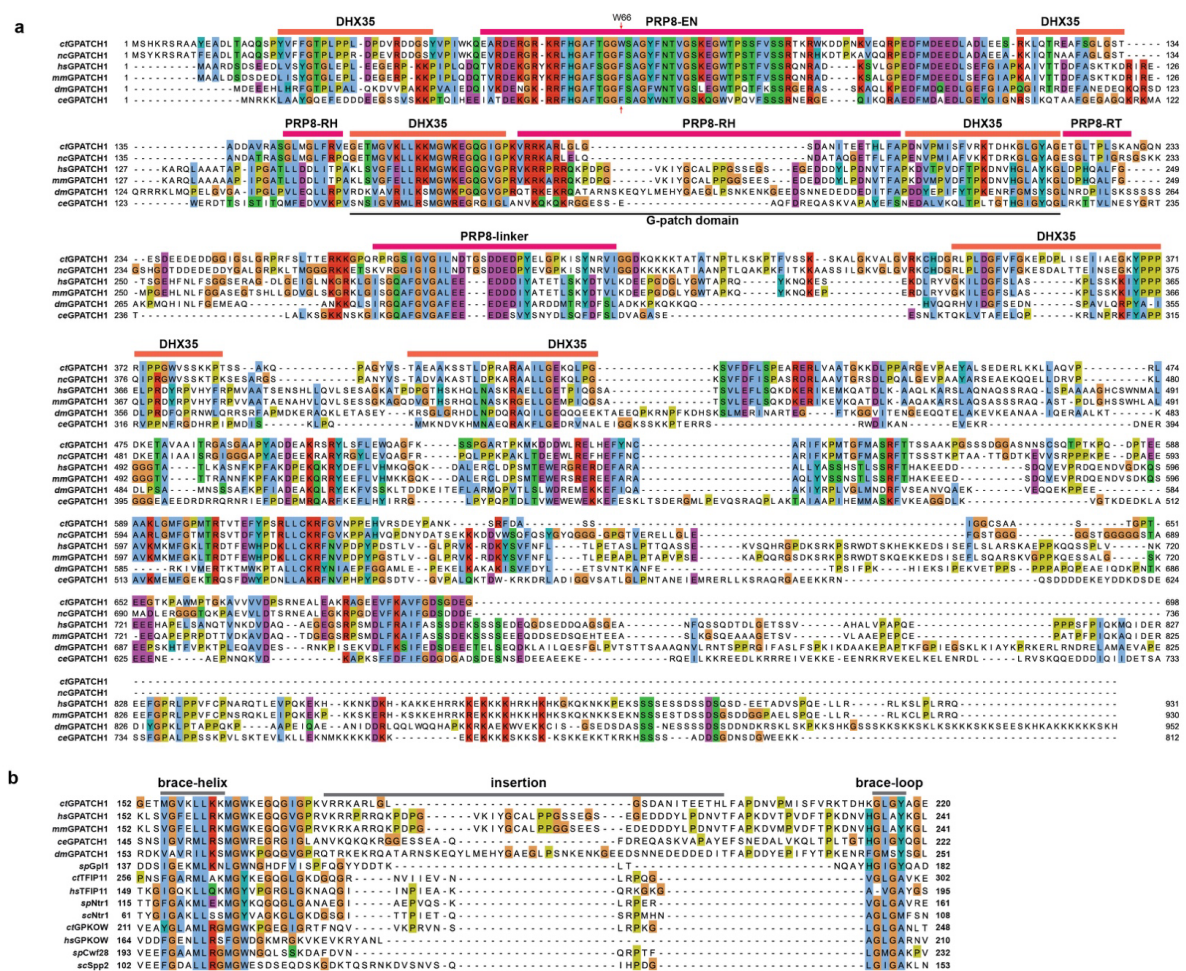

Supplement: Supplementary file 11 — Supplementary information, Figure S11 [file 41422_2025_1084_MOESM11_ESM.pdf]

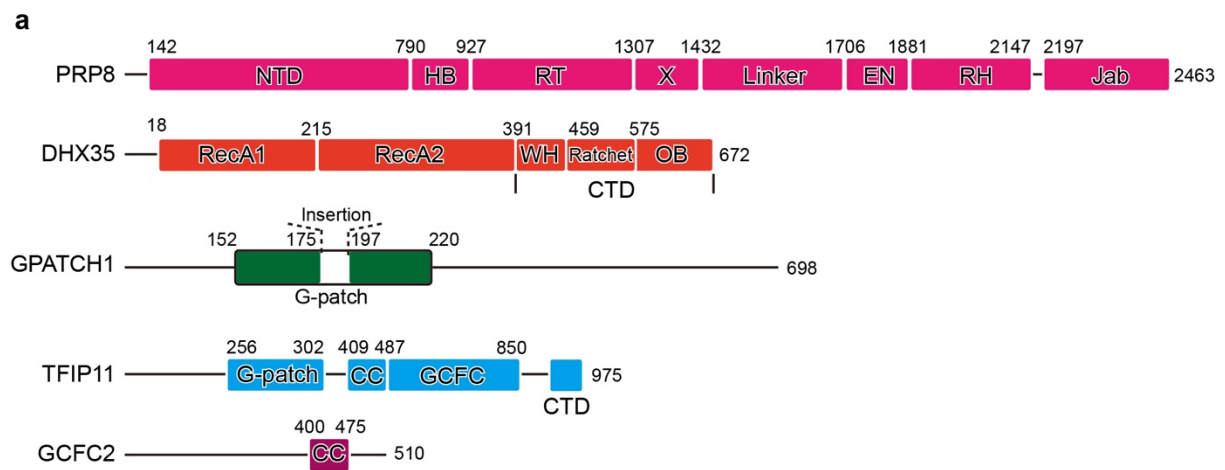

**Figure S12: Domain architecture of key splicing components from *C. thermophilum*.**

Supplement: Supplementary file 12 — Supplementary information, Figure S12 [file 41422_2025_1084_MOESM12_ESM.pdf]
