# Supplementary material for: Structural insights into spliceosome fidelity: DHX35–GPATCH1- mediated rejection of aberrant splicing substrates
Source: Cell Res. 2025 Feb 28;35(4):296–308. doi: 10.1038/s41422-025-01084-w (PMC11958768; doi:10.1038/s41422-025-01084-w)
Supplement: Supplementary file 13 — Supplementary information, Figure S13 [file 41422_2025_1084_MOESM13_ESM.pdf]

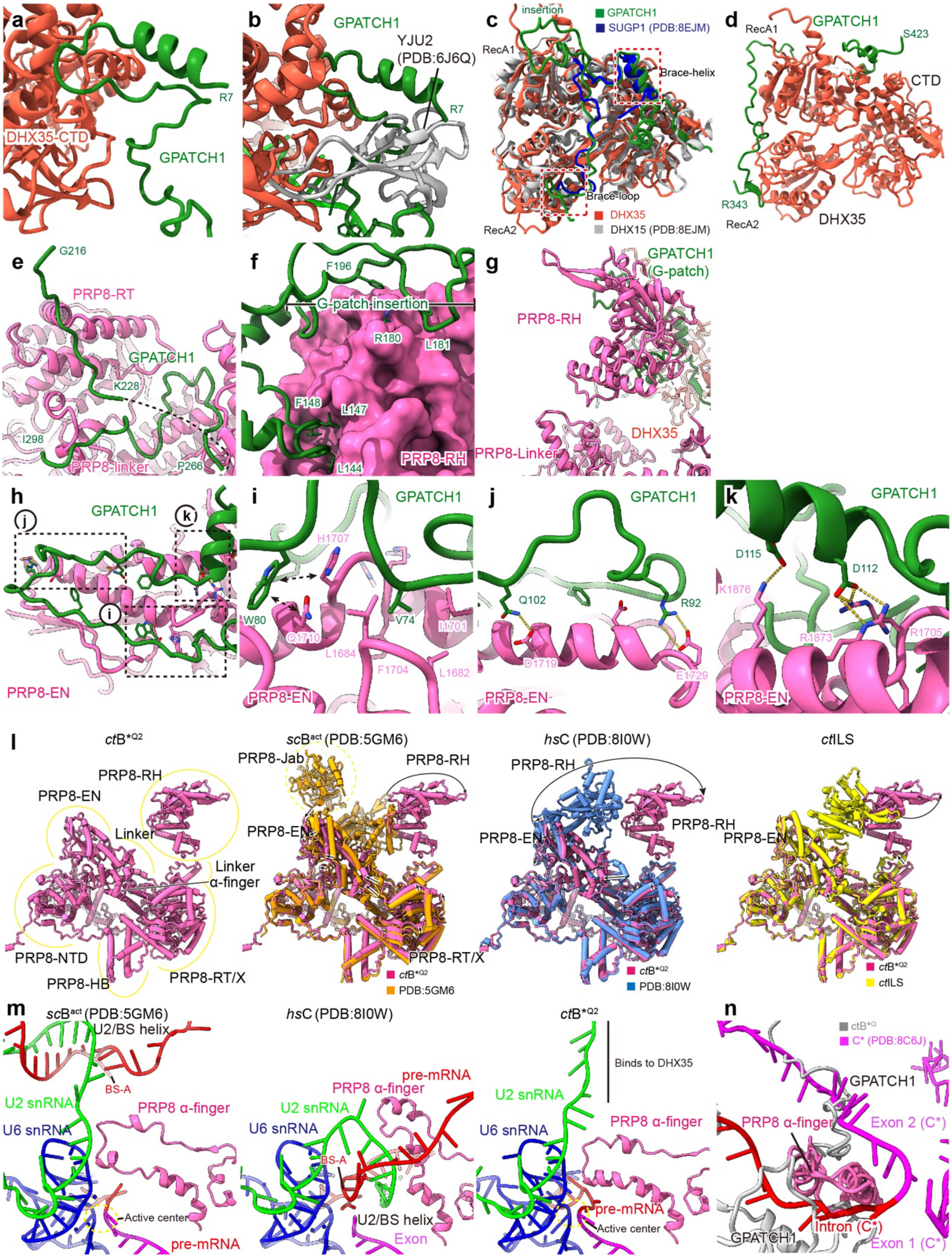

Figure S13: Molecular details of GPATCH1 binding to DHX35 and PRP8

a, The N-terminus of GPATCH1 binds to the C-terminal domain of DHX35 (DHX35-CTD). **b**, The region where GPATCH1 binds to DHX35 in *ct*B*^{Q2} spatially clashes with the binding site of YJU2 in the *sc*B* complex (PDB: 6J6Q). **c**, Superimposition of the DHX35 (tomato) and GPATCH1 (forest green) complex with the crystal structure of

DHX15 (gray) and SUGP1 (blue) (PDB: 8EJM). **d**, The middle region of GPATCH1 (aa 343-423) wraps around DHX35. **e**, A small stretch following the G-patch domain (aa 220-298) binds to the RT and linker domain of PRP8. **f**, the G-patch insertion forms interactions with the PRP8-RH domain. **g**, In the B^{*Q} complex, the PRP8-RH domain adopts a position close to the PRP8-linker. **h-k**, interactions between GPATCH1 and PRP8-EN. **l**, Rearrangements of the PRP8 domains during B^{act} to ILS transitions. **m**, The PRP8 a-finger is rearranged from B^{act} to C to accommodate the relocation of the U2/BS helix (left to middle panel). In B^{*Q2}, the α-finger adopts a conformation that more closely resembles its position in the B^{act} complex. **n**, Superimposition of GPATCH1 (*ct*B^{*Q2}) and the catalytic center of the C* complex (PDB: 8C6J) depicting clashes between the pre-mRNA and GPATCH1.
