# Supplementary material for: Structural insights into spliceosome fidelity: DHX35–GPATCH1- mediated rejection of aberrant splicing substrates
Source: Cell Res. 2025 Feb 28;35(4):296–308. doi: 10.1038/s41422-025-01084-w (PMC11958768; doi:10.1038/s41422-025-01084-w)
Supplement: Supplementary file 14 — Supplementary information, Figure S14 [file 41422_2025_1084_MOESM14_ESM.pdf]

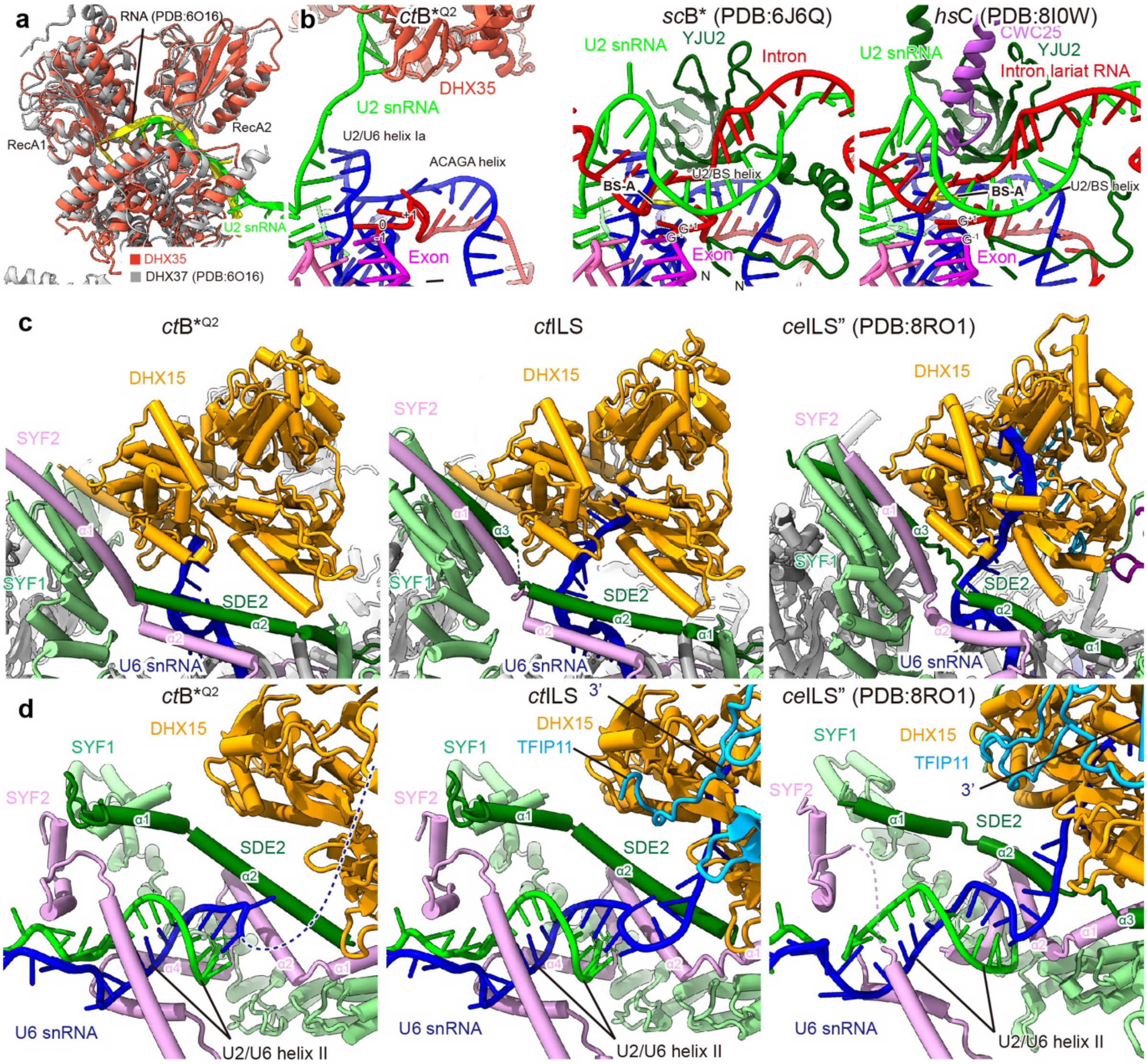

Figure S14: Structural details of the *ct*B*^{Q2} state.

a, Superimposition of DHX35 (tomato red) with the crystal structure of nucleotide-free DHX37 (gray) bound to RNA (yellow, PDB: 6O16). U2 snRNA from *ct*B*^{Q2} is indicated in green. **b**, Structural comparison of the splicing active center and U2/BS duplex with surrounding factors in *ct*B*^{Q2} (left), *sc*B* (middle, PDB: 6J6Q), and *hs*C (right, PDB: 8I0W). In the *ct*B*^{Q2} complex, the U2 snRNA is engaged with DHX35, and the BS has already been released. In the further progressed B* and C complexes, YJU2 and CWC25 stabilize the U2/BS helix during the first step of splicing. **c**, cryo-EM model of the docking site of DHX15 in the *ct*B*^{Q2} (left), *ct*ILS (middle) and *ce*ILS" (PDB:8RO1, right) complexes. Highlighted components: DHX15 (orange), SYF2 (pink), SDE2 (green), SYF1 (light green) and CWF19L (purple). The U6 snRNA is shown in blue. **d**, Comparison of U2/U6 helix II in relation to DHX15 in *ct*B*^{Q2}, *ct*ILS and *ce*ILS'' (PDB: 8RO1). SYF2 and SDE2 form similar interactions, wrapping around the U2/U6 helix II.
