# Supplementary material for: Structural insights into spliceosome fidelity: DHX35–GPATCH1- mediated rejection of aberrant splicing substrates
Source: Cell Res. 2025 Feb 28;35(4):296–308. doi: 10.1038/s41422-025-01084-w (PMC11958768; doi:10.1038/s41422-025-01084-w)
Supplement: Supplementary file 15 — Supplementary information, Tables S1 [file 41422_2025_1084_MOESM15_ESM.pdf]

Table S1. *C. thermophilum* homolog of the human splicing register. Ortholog genes were identified using the annotation of different databases as described in Methods. Commonly used aliases are highlighted in bold.

Homo sapiens				Chaetomium thermophilum		Saccharomyces cerevisiae		
gene ID	protein ID	protein name	alias	gene ID	protein ID	gene ID	Protein ID	protein name
U1 snRNP								
HGNC:11150	P08621	**SNRP70**	**U1-70K**, RPU1, SNRP70, U1AP1	**CTHT_0025670**	G0S615	YIL061C	Q00916	Snp1
HGNC:11151	Q8WTU0	**SNRPA**	Mud1, **U1-A**, U1A	**CTHT_0033900**	G0S622	YBR119W	P32605	Mud1
HGNC:11157	P09234	**SNRPC**	**U1C**, Yhc1, U1-C	**CTHT_0051860**	G0SDI0	YLR298C	Q05900	Yhc1
Sm proteins								
HGNC:11153	P14678	**SNRPB**	COD, **SMB/SB'**, snRNP-B, SNRPB1	**CTHT_0072980**	G0SHQ3	YER029C	P40018	Smb1
HGNC:11158	P62314	**SNRPD1**	**SMD1**, SNRPD, HsT2456	**CTHT_0003330**	G0RZL0	YGR074W	Q02260	Smd1
HGNC:11162	P62306	**SNRPF**	**SMF**	**CTHT_0005760**	G0RY82	YPR182W	P54999	Smx3
HGNC:11161	P62304	**SNRPE**	**SME**	**CTHT_0038260**	G0S8J0	YOR159C	Q12330	Sme1
HGNC:11160	P62318	**SNRPD3**	**SMD3**, Sm-D3	**CTHT_0065260**	G0SG70	YLR147C	P43321	Smd3
HGNC:11163	P62308	**SNRPG**	**SMG**	**CTHT_0074540**	G0SI53	YFL017W-A	P40204	Smx2
HGNC:11159	P62316	**SNRPD2**	**SMD2**, SNRPD1	**CTHT_0022090**	G0S405	YLR275W	Q06217	Smd2
Lsm proteins								
HGNC:13940	Q9Y333	**LSM2**	C6orf28, G7b, YBL026W	**CTHT_0003570**	G0RZN3	YBL026W	P38203	LSm2
HGNC:20472	O15116	**LSM1**	LSM1, CASM, YJL124C	**CTHT_0005870**	G0RY92	YJL124C	P47017	Lsm1
HGNC:20470	Q9UK45	**LSM7**	YNL147W	**CTHT_0011670**	G0S0Y4	YNL147W	P53905	Lsm7
HGNC:17259	Q9Y4Z0	**LSM4**	YER112W, GRP	**CTHT_0032950**	G0S5H2	YER112W	P40070	Lsm4
HGNC:20471	O95777	**LSM8**	YJR022W, NAA38	**CTHT_0060100**	G0SEY1	YJR022W	P47093	Lsm8
HGNC:17874	P62310	**LSM3**	YLR438C, SMX4, USS2	**not annotated**	NA	YLR438C-A	P57743	Lsm3
HGNC:17162	Q9Y4Y9	**LSM5**	YER146W	**-**	-	YER146W	P40089	Lsm5
HGNC:17017	P62312	**LSM6**	YDR378C	**not annotated**	NA	YDR378C	Q06406	Lsm6
U2 snRNP								
HGNC:11152	P09661	**SNRPA1**	**U2A**, Lea1	**CTHT_0034680**	G0S6F0	YPL213W	Q08963	Lea1
HGNC:11155	P08579	**SNRPB2**	U2snRNPB, Msl1, **U2B"**	**CTHT_0054050**	G0SBL9	YIR009W	P40567	Msl1
HGNC:18000	Q7RTV0	**PHF5A**	SFSB7, SAP14b, Rds3, INI	**not annotated**	NA	YPR094W	Q06835	Rds3
HGNC:10765	Q15459	**SF3A1**	SF3a120, SAP114, PRPF21, Prp21	**CTHT_0011550**	G0S0X2	YJL203W	P32524	Prp21
HGNC:10766	Q15428	**SF3A2**	SAP62, PRPF11, SF3a66, Prp11	**CTHT_0034960**	G0S6H7	YDL043C	Q07350	Prp11
HGNC:10767	Q12874	**SF3A3**	SAP61, SF3a60, PRPF9, Prp9	**CTHT_0027610**	G0S767	YDL030W	P19736	Prp9
HGNC:10768	O75533	**SF3B1**	SAP155, PRPF10, Prp10, Hsh155	**CTHT_0003510**	G0RZM7	YMR288W	P49955	Hsh155
HGNC:10769	Q13435	**SF3B2**	SAP145, SF3b145, SF3b1	**CTHT_0006280**	G0RYD3	YMR240C	Q02554	Cus1
HGNC:10770	Q15393	**SF3B3**	SAP130, SF3b130, RSE1, KIAA0017	**CTHT_0024090**	G0S5A2	YML049C	Q04693	Rse1
HGNC:10771	Q15427	**SF3B4**	SAP49, SF3b49, Hsh49	**CTHT_0061980**	G0SE06	YOR319W	Q99181	Hsh49
HGNC:21083	Q9BWJ5	**SF3B5**	SF3b10, MGC3133, Ysf3	**CTHT_0012180**	G0S133	YNL138W-A	P0C074	Ysf3
HGNC:30096	Q9Y3B4	**SF3B6**	HSPC175, P14, SAP14, SF3B14a	**CTHT_0054490**	G0SBR3	-	-	-
U5 snRNP								
HGNC:17340	Q6P2Q9	**PRPF8**	PRPC8, RP13, **PRP8**, SNRNP220	**CTHT_0071250**	G0SFL3	YHR165C	P33334	Prp8
HGNC:30859	O75643	**SNRNP200**	RP33, U5-200KD, KIAA0788, **BRR2**	**CTHT_0009470**	G0S0B9	YER172C	P32639	Brr2
HGNC:30858	Q15029	**EFTUD2**	U5-116KD, **SNU114**, SNRNP116	**CTHT_0054900**	G0SBV2	YKL173W	P36048	Snu114
HGNC:30857	Q96DI7	**SNRNP40**	**U5-40kD**, 40K, FLJ41108, PRP8BP	**CTHT_0047230**	G0S9U7	-	-	-
HGNC:15860	O94906	**PRPF6**	C20ORF14, **PRP6**, SNRNP102, U5-102K	**CTHT_0051060**	G0SDA3	YBR055C	P19735	Prp6
HGNC:30551	P83876	**TXNL4A**	U5-15K, DIB1, **DIM1**, U5-15kD	**CTHT_0027370**	G0S743	YPR082C	Q06819	Dib1
HGNC:26041	Q9NX01	**TXNL4B**	Dim1-like protein, **DIM2**, DLP, FLJ20511	**CTHT_0027370**	G0S743	YPR082C	Q06819	Dib1
HGNC:17347	Q9BUQ8	**DDX23**	**PRP28**, U5-100K, PRPF28, SNRNP100	**CTHT_0054430**	G0SBQ7	YDR243C	P23394	Prp28
U4/U6								
HGNC:17348	O43395	**PRPF3**	RP18, **PRP3**, hPrp3, SNRNP90	**CTHT_0002950**	G0RZH2	YDR473C	Q03338	Prp3
HGNC:17349	O43172	**PRPF4**	**PRP4**, HPRP4, HPRP4P, SNRNP60	**CTHT_0054790**	G0SBU2	YPR178W	P20053	Prp4
HGNC:15446	Q8WWY3	**PRPF31**	RP11, **PRP31**, SNRNP61	**CTHT_0010050**	G0S0H6	YGR091W	P49704	Prp31
HGNC:7819	P55769	**SNU13**	SSFA1, NHP2L1, SNRNP15-5, 15.5K	**CTHT_0066810**	G0SGM0	YEL026W	P39990	Snu13
HGNC:14651	O43447	**PPIH**	CYP20, CYPH, USA-CYP, MGC5016	**CTHT_0058210**	G0SCS0	-	-	-
U4/U6.U5								
HGNC:10538	O43290	**START1**	**SNU66**, SNRNP110, HAF, Ara1	**CTHT_0002250**	G0RZA3	YOR308C	Q12420	Snu66
HGNC:20071	Q53GS9	**USP39**	**SAD1**, SNRNP65, CGI-	**CTHT_0065550**	G0SG97	YFR005C	P43589	Sad1
HGNC:30240	Q8WVK2	**SNRNP27**	RY1, U4/U6.U5-27K	**CTHT_0028280**	G0S7I7	-	-	-
HGNC:28117	Q9BTD8	**RBM42**	MGC10433	**CTHT_0060260**	G0SEZ7	-	-	-
HGNC:17346	Q13523	**PRP4K**	**PRP4**, PRP4H, KIAA0536, PRP4 kinase	**CTHT_0030760**	G0S455	-	-	-

Homo sapiens				Chaetomium thermophilum		Saccharomyces cerevisiae		
gene ID	protein ID	protein name	alias	gene ID	protein ID	gene ID	Protein ID	protein name
**U1-related**								
HGNC:16463	O75400	**PRPF40A**	FNBP3, FLJ20585, FBP11, HIP10, PRP40	**CTHT_0021190**	G0S8B9	YPR152C	Q06525	Urn1
HGNC:15923	Q14498	**RBM39**	CAPERalpha, FLJ44170, HCC1, RNPC2	**CTHT_0006960**	G0RYJ9	-	-	-
HGNC:23244	P49756	**RBM25**	RNPC7, S164, Snu71, fSAP94	**CTHT_0035420**	G0S6S4	YGR013W	P53207	Snu71
HGNC:21608	Q9Y383	**LUC7L2**	LUC7L2, FLJ10657, LUC7B2	**CTHT_0064370**	G0SEN4	YDL087C	Q07508	Luc7
HGNC:24309	O95232	**LUC7L3**	FLJ11063, CRA, LUC7A, OA48-18	**-**	-	-	-	-
**U2-related**								
HGNC:18681	Q7L014	**DDX46**	Prp5, PRPF5, KIAA0801, FLJ25329	**CTHT_0009560**	G0S0C8	YBR237W	P21372	Prp5
HGNC:5276	O43719	**HTATSF1**	TAT-SF1	**CTHT_0057890**	G0SCN8	YNL286W	P53830	Cus2
HGNC:16900	O75940	**SMNDC1**	SMNR, **SPF30**, TDRD16C	**CTHT_0042500**	G0SAJ4	-	-	-
HGNC:17042	Q9UHX1	**PUF60**	FIR, FLJ31379, RoBPI, SIAHBP1	**-**	-	-	-	-
HGNC:30855	O15042	**U2SURP**	fSAPa, SR140	**CTHT_0042700**	G0SAL4	-	-	-
HGNC:12453	Q01081	**U2AF1**	DKFZp313J1712, RNU2AF1, U2AF35	**CTHT_0056140**	G0SC69	-	-	-
HGNC:23156	P26368	**U2AF2**	U2AF65	**CTHT_0003520**	G0RZM8	YKL074C	P36084	Mud2
HGNC:18676	Q86XP3	**DDX42**	FLJ43179, RHELP, RNAHP, SF3b125	**-**	-	-	-	-
**B-specific**								
HGNC:26433	Q96NC0	**ZMAT2**	**SNU23**, FLJ31121	**CTHT_0035280**	G0S6R0	YDL098C	Q12368	Snu23
HGNC:5958	Q13123	**IK**	**RED**, RER	**not annotated**	NA	-	-	-
HGNC:12739	O75554	**WBP4**	**FBP21**, FNBP21, MGC117310	**CTHT_0063990**	G0SEJ7	-	-	-
HGNC:16461	Q9Y2W2	**WBP11**	NPWBP, SIPP1, SNP70, **NPW38BP**	**CTHT_0012760**	G0S191	YBR194W	P38305	AIM4
HGNC:9330	O60828	**PQBP1**	**NPW38**, RENS1, MRXS8, SHS, MRX55	**-**	-	-	-	-
HGNC:25930	Q8NAV1	**PRPF38A**	FLJ14936, **PRP38A**	**CTHT_0013190**	G0S1D3	YGR075C	Q00723	Prp38
HGNC:7032	P55081	**MFAP1**	AMP	**CTHT_0069660**	G0SHD7	YBR152W	P38282	Spp381
HGNC:18247	Q2TAY7	**SMU1**	fSAP57, sSMU-1, FLJ10805, BWD	**-**	-	-	-	-
HGNC:13736	Q9BZL1	**UBL5**	UBL5p, FLJ46917, HUB1, MGC131795	**CTHT_0006510**	G0RYF5	YNR032C-A	Q6Q546	Hub1
**PRP19/CDC5L**								
HGNC:17896	Q9UMS4	**PRPF19**	PSO4, SNEV, **PRP19**, NMP200, UBOX4	**CTHT_0072540**	G0SFY0	YLL036C	P32523	Prp19
HGNC:1743	Q99459	**CDC5L**	CDC5, CEF1, PCDC5RP	**CTHT_0038140**	G0S8A6	YMR213W	Q03654	Cef1
HGNC:9089	O43660	**PLRG1**	PRL1, PRPF46, Prp46, Cwc1, TANGO4	**CTHT_0008910**	G0S066	YPL151C	Q12417	Prp46
HGNC:975	O75934	**BCAS2**	**SPF27**, DAM1, Snt309	**CTHT_0052340**	G0SDM8	YPR101W	Q06091	SNT309
HGNC:26939	Q9P013	**CWC15**	HSPC148, C11orf5, Cwf15, AD-002	**CTHT_0027480**	G0S754	YDR163W	Q03772	Cwc15
HGNC:15879	Q8WYA6	**CTNNBL1**	C20orf33	**CTHT_0045540**	G0S9E3	-	-	-
HGNC:5241	P11142	**HSPA8**	NIP71, HSPA10, HSP70, HSC54, **HSP7C**	**CTHT_0007430**	G0RYP6	YAL005C	P10591	SSA1
**IBC**								
HGNC:9258	Q9UNP9	**PPIE**	CYP33, CyP-33, MGC3736, CypE	**CTHT_0002650**	G0RZE2	-	-	-
HGNC:14089	Q9HCS7	**XAB2**	HCRN, HCNP, **SYF1**, NTC90	**CTHT_0033880**	G0S620	YDR416W	Q04048	Syf1
HGNC:29201	Q9ULR0	**ISY1**	KIAA1160, fSAP33	**CTHT_0008120**	G0RZZ0	YJR050W	P21374	Isy1
HGNC:37286	A6NGH7	**CCDC16**	ZNF830, MGC20398, OMCG1, SEL13	**-**	-	-	-	-
HGNC:29513	O60306	**AQR**	DKFZp686B23123, IBP160, KIAA0560	**CTHT_0071530**	G0SFN9	-	-	-
**PRP19/CDC5L related**								
HGNC:16696	Q13573	**SNW1**	PRPF45, Prp45, **SKIP**, SKIP1, FUN20	**CTHT_0061180**	G0SF87	YAL032C	P28004	Prp45
HGNC:15762	Q9BZJ0	**CRNKL1**	CRN, CLF, **SYF3**, Clf1	**CTHT_0010060**	G0S0H7	YLR117C	Q12309	Syf3
HGNC:9260	Q9Y3C6	**PPIL1**	CYPL1	**CTHT_0014950**	G0S1V3	-	-	-
HGNC:25503	Q9NW64	**RBM22**	ZC3H16, Cwc2, fSAP47, FLJ10290	**CTHT_0028670**	G0S7S7	YDL209C	Q12046	Cwc2
HGNC:29629	P41223	**BUD31**	G10, EDG2, Cwc14, fSAP17, YCR063W	**CTHT_0023130**	G0S4Q2	YCR063W	P25337	Bud31
HGNC:9343	Q92733	**PRCC**	RCCP1	**CTHT_0024450**	G0S5D8	-	-	-
HGNC:19824	O95926	**SYF2**	GCIPIP, p29, DKFZp564O2082, GCIP	**CTHT_0024880**	G0S5N3	YGR129W	P53277	Syf2
HGNC:25503	Q9NW64	**RBM22**	ZC3H16, Cwc2, fSAP47, FLJ10290	**CTHT_0006330**	G0RYD8	YDL209C	Q12046	Ecm2
**Bact proteins**								
HGNC:29322	Q9HCG8	**CWC22**	KIAA1604, EIF4GL, fSAPb, NCM	**CTHT_0025210**	G0S5W9	YGR278W	P53333	Cwc22
HGNC:17350	O60508	**CDC40**	PRPF17, EHB3, Prp17	**CTHT_0017830**	G0S2N1	YDR364C	P40968	Prp17
HGNC:16639	Q9UQ35	**SRRM2**	SMD2D, SRL300, KIAA0324, Cwc21	**CTHT_0022970**	G0S4I7	YDR482C	Q03375	Cwc21
HGNC:28332	Q8WUD4	**CCDC12**	MGC23918	**CTHT_0052020**	G0SDJ6	-	-	-
HGNC:10664	Q6UX04	**CWC27**	SDCCAG10, NY-CO-10	**CTHT_0005290**	G0RY38	YPL064C	Q02770	Cwc27
HGNC:12974	O15541	**RNF113A**	ZNF183, RNF113, CWC24	**CTHT_0011920**	G0S108	YLR323C	P53769	Cwc24
HGNC:9261	Q13356	**PPIL2**	CYP6, CYC4, Cyp-60, UBOX7	**CTHT_0070810**	G0SFH2	-	-	-
HGNC:16638	Q8IYB3	**SRRM1**	SRM160, POP101, MGC39488	**CTHT_0056660**	G0SCB8	-	-	-

Homo sapiens				Chaetomium thermophilum		Saccharomyces cerevisiae		
gene ID	protein ID	protein name	alias	gene ID	protein ID	gene ID	Protein ID	protein name
**Step I factors**								
HGNC:30677	Q92917	**GPKOW**	T54, GPATC5, GPATCH5, Spp2	**CTHT_0071470**	G0SFN3	YOR148C	Q02521	Spp2
HGNC:25989	Q9NXE8	**CWC25**	CCDC49, FLJ20291, DKFZp779M0968	**CTHT_0001190**	G0RZ02	YNL245C	P53854	Cwc25
HGNC:25518	Q9BW85	**YJU2**	CCDC94, FLJ10374	**CTHT_0065640**	G0SGA6	YKL095W	P28320	Yju2
HGNC:2739	O60231	**DHX16**	DBP2, DDX16, Prp2, PRPF2, PRP8	**CTHT_0063660**	G0SEG4	YNR011C	P20095	Prp2
**RES**								
HGNC:24282	Q9Y388	**RBMX2**	CGI-79, **SNU17**	**CTHT_0011020**	G0S0S0	YIR005W	P40565	Snu17
HGNC:30587	Q8TAD8	**SNIP1**	PML1	**CTHT_0017490**	G0S2J9	YLR016C	Q07930	Pml1
HGNC:28199	Q9BRD0	**BUD13**	Cwc26; fSAP71, MGC13125	**CTHT_0029350**	G0S847	YGL174W	P46947	Bud13
**EJC**								
HGNC:18683	P38919	**eIF4A3**	DDX48, KIAA0111, EIF4AIII	**CTHT_0027620**	G0S768	YDR021W	Q12099	Fal1
HGNC:9905	Q9Y5S9	**RBM8A**	**Y14**, ZNRP, BOV-1A, RBM8B	**CTHT_0073330**	G0SHT8	-	-	-
HGNC:6815	P61326	**MAGOH**	MAGOHA, MAGOH1	**not annotated**	NA	-	-	-
HGNC:17040	O15234	**CASC3**	ML51, BTZ, MLN51	**CTHT_0062830**	G0SE83	-	-	-
HGNC:13917	Q13838	**DDX39B**	BAT1, **UAP56**, p47	**CTHT_0012740**	G0S189	YDL084W	Q07478	Sub2
HGNC:9162	Q9H307	**PNN**	Pinin, DRS, DRSP, SDK3, memA	**CTHT_0024660**	G0S5L3	-	-	-
**C proteins**								
HGNC:3954	Q14331	**FRG1**	FSG1, FRG1A	**CTHT_0054990**	G0SBW1	-	-	-
HGNC:28954	Q96BP3	**PPWD1**	KIAA0073, CyP64	**CTHT_0059490**	G0SES0	-	-	-
HGNC:15702	Q8WUA2	**PPIL4**	HDCME13P	**CTHT_0041950**	G0SAE1	-	-	-
HGNC:25305	Q9H0G5	**NSRP1**	CCDC55, DKFZp434K1421, FLJ37233	**CTHT_0023730**	G0S515	-	-	-
HGNC:25495	Q9NW82	**WDR70**	FLJ10233	**not annotated**	NA	-	-	-
HGNC:17211	Q92620	**DHX38**	**PRP16**, DDX38, PRPF16, KIAA0224	**CTHT_0009880**	G0S0F9	YKR086W	P15938	Prp16
HGNC:24217	Q86X95	**CIR1**	CIR	**-**	-	-	-	-
HGNC:10288	Q8TA86	**RP9**	PAP1	**-**	-	-	-	-
**Step II factors**								
HGNC:16939	O95391	**SLU7**	hSLU7, 9G8	**CTHT_0011780**	G0S0Z5	YDR088C	Q02775	Slu7
HGNC:1735	Q16543	**PRP18**	FLJ10210, PRPF18, hPrp18	**CTHT_0024370**	G0S5D0	YGR006W	P33411	Prp18
HGNC:2749	Q14562	**DHX8**	DDX8, **PRP22**, PRPF22, HRH1, Dhr2	**CTHT_0035640**	G0S700	YER013W	P24384	Prp22
**C* proteins**								
HGNC:9262	Q9H2H8	**PPIL3**	CypJ	**CTHT_0005270**	G0RY36	-	-	-
HGNC:14650	Q13427	**PPIG**	CASP10, SCAF10, CypG	**-**	-	-	-	-
HGNC:24563	Q9Y421	**FAM32A**	DKFZP586O0120, OTAG-12	**CTHT_0061400**	G0SFA9	-	-	-
HGNC:16817	Q96DF8	**ESS2**	DGCR14, DGCR13, bis1, ESS-2, Es2el	**CTHT_0067690**	G0SGV3	-	-	-
HGNC:15502	Q96BZ8	**LENG1**		**CTHT_0006650**	G0RYG9	-	-	-
HGNC:17946	Q9Y314	**NOSIP**	CGI-25	**CTHT_0026000**	G0S6A0	-	-	-
HGNC:24932	Q9NZ63	**C9orf78**	HCA59, CSU2, **TLS1**	**CTHT_0009850**	G0S0F6	-	-	-
HGNC:29873	Q8N5F7	**NKAP**	FLJ22626, MRXSHD	**-**	-	-	-	-
HGNC:29938	Q8WUQ7	**Cactin**	C19orf29, NY-REN-24	**CTHT_0074270**	G0SI28	-	-	-
HGNC:18786	Q14320	**FAM50A**	DXS9928E, XAP5, HXC-26, 9F	**CTHT_0031710**	G0S4U5	-	-	-
HGNC:18789	Q9Y247	**FAM50B**	D6S2654E, X5L	**CTHT_0031710**	G0S4U5	-	-	-
HGNC:21894	Q9H875	**PRKRIP1**	C114, FLJ13902, KRBOX3	**CTHT_0002440**	G0RZC1	-	-	-
HGNC:18674	Q9UJV9	**DDX41**	DHX41, ABS, MGC8828, **Abstrakt**	**-**	-	-	-	-
HGNC:26239	Q9H5V9	**STEEP1**	FLJ22965, **CXorf56**	**-**	-	-	-	-
HGNC:26643	Q6IQ49	**SDE2**	C1orf55, FLJ35382	**CTHT_0002620**	G0RZD9	-	-	-
HGNC:15861	Q9H5Z1	**DHX35**	C20orf15, DDX35	**CTHT_0002590**	G0RZD6	-	-	-
HGNC:24658	Q9BRR8	**GPATCH1**	GPATC1, ECGP, FLJ10206, FLJ3868	**CTHT_0041140**	G0SA63	-	-	-
HGNC:32672	Q9BRX9	**WDR83**	**MORG1**	**CTHT_0065610**	G0SGA3	-	-	-
HGNC:1162	Q70Z53	**FRA10AC1**	C10orf4, F26C11.1-like, FRA10A	**-**	-	-	-	-
HGNC:24697	Q96N46	**TTC14**	DKFZp313M1015, DRDL5813, FLJ00166	**-**	-	-	-	-
**Disassembly**								
HGNC:2738	O43143	**DHX15**	DBP1, DDX15, PRPF43, PRP43, HRH2	**CTHT_0005780**	G0RY84	YGL120C	P53131	Prp43
HGNC:17165	Q9UBB9	**TFIP11**	TIP39, Spp382, DKFZP434B194, Ntr1	**CTHT_0020180**	G0S394	YLR424W	Q06411	Ntr1
HGNC:25613	Q69YN2	**CWF19L1**	FLJ13922, FLJ10998, RP11-316M21.3	**CTHT_0072860**	G0SHP4	YGR093W	P53255	Drn1
HGNC:26508	Q2TBE0	**CWF19L2**	FLJ32343	**CTHT_0035950**	G0S730	-	-	-
HGNC:1317	P16383	**GCFC2**	C2orf3, DNABF, GCF, TCF9	**CTHT_0074550**	G0SI54	YKR022C	P36118	Ntr2
HGNC:13579	Q9Y5B6	**PAXBP1**	C21orf66, FLJ90561, GCFC, GCFC1	**CTHT_0074550**	G0SI54	YKR022C	P36118	Ntr2

Homo sapiens				Chaetomium thermophilum		Saccharomyces cerevisiae		
gene ID	protein ID	protein name	alias	gene ID	protein ID	gene ID	Protein ID	protein name
**Other splicing factors**								
HGNC:28118	P13994	**YJU2B**	MGC10471	**CTHT_0040600**	G0SA10	-	-	-
HGNC:20155	Q86U06	**RBM23**	CAPERbeta, FLJ10482, RNPC4, SF2	**CTHT_0006960**	G0RYJ9	-	-	-
HGNC:25031	Q6NWY9	**PRPF40B**	HYPC	**CTHT_0059960**	G0SEW7	YPR152C	Q06525	Urn1
HGNC:2985	Q14183	**FAM192A**	NIP30, C16orf94, FLJ21799, PSME3IP1	**CTHT_0034990**	G0S6N3	-	-	-
HGNC:9903	P78332	**RBM6**	DEF3, DKFZp686B0877, FLJ36517	**CTHT_0014120**	G0S1M4	-	-	-
HGNC:23020	Q8WU68	**U2AF1L4**	FLJ35525, U2AF1L3, U2af26	**CTHT_0056140**	G0SC69	-	-	-
HGNC:12950	Q15637	**SF1**	ZNF162, ZFM1, ZCCHC25	**CTHT_0068240**	G0SH06	YLR116W	Q12186	Msl5
HGNC:9896	P98175	**RBM10**	DXS8237E, FLJ40796, GPATCH9	**CTHT_0014120**	G0S1M4	-	-	-
HGNC:9902	P52756	**RBM5**	FLJ39876, G15, H37, LUCA15, RMB5	**CTHT_0014120**	G0S1M4	-	-	-
HGNC:24101	Q9BXP5	**SRRT**	**ARS2B**, ASR2, MGC126427	**CTHT_0054450**	G0SBQ9	-	-	-
HGNC:1770	Q15131	**CDK10**	ALSAS, PISSLRE	**-**	-	-	-	-
HGNC:19032	Q9BU76	**C1orf35**	MGC4174, **MMTAG2**	**CTHT_0067540**	G0SGT9	-	-	-
HGNC:8014	P67809	**YBX1**	CSDB, DBPB, EFI-A, NSEP1, **YBOX1**	**-**	-	-	-	-
HGNC:25954	Q8TBK6	**ZCCHC10**	FLJ20094	**not annotated**	NA	-	-	-
HGNC:3312	Q15717	**ELAVL1**	**ELAV1**, Hua, HUR, MelG	**-**	-	-	-	-
HGNC:2428	P16989	**YBX3**	CSDA, DBPA, **YBOX3**	**-**	-	-	-	-
HGNC:2326	Q9UKF6	**CPSF3**	CPSF 73kD, CPSF, CPSF73, YSH1	**CTHT_0006720**	G0RYH6	-	-	-
HGNC:18643	Q8IWZ8	**SUGP1**	DKFZp434E2216, F23858, RBP, SF4	**-**	-	-	-	-
HGNC:28291	Q96NB3	**ZNF830**	CCDC16, MGC20398, OMCG1, SEL13	**CTHT_0064280**	G0SEM5	-	-	-
HGNC:6327	O60870	**KIN**	HsKin17, BTCD, KIN17	**CTHT_0067770**	G0SGW1	YOR077W	P40962	Rts2
HGNC:20314	Q86UA1	**PRPF39**	PRP39, FLJ45460, MGC149842	**CTHT_0054140**	G0SBM8	YML046W	P39682	Prp39
HGNC:16944	Q96I25	**RBM17**	DKFZp686F13131, MGC14439, SPF45	**CTHT_0006960**	G0RYJ9	-	-	-
HGNC:29243	Q9P2N5	**RBM27**	ARRS1, KIAA1311, Psc1	**CTHT_0033710**	G0S5V0	-	-	-
HGNC:20327	Q5T8P6	**RBM26**	ARRS2, C13orf10, FLJ20957	**CTHT_0033710**	G0S5V0	-	-	-
HGNC:25556	Q9NVM6	**DNAJC17**	FLJ10634	**CTHT_0070640**	G0SHN2	YGL128C	P52868	Cwc23
HGNC:22935	Q92797	**SYMPK**	BAG-1, HAP, RAP46	**CTHT_0073250**	G0SHT0	YAL043C	Q01329	Pta1
HGNC:15594	Q9UK59	**DBR1**	XGIP	**CTHT_0006420**	G0RYE6	YKL149C	P24309	Dbr1
HGNC:25512	Q5VTL8	**PRPF38B**	FLJ10330, MGC163218, NET1	**CTHT_0070610**	G0SHM9	-	-	-
**CAP binding proteins**								
HGNC:7659	P52298	**NCBP2**	CBC2, CBP20, NIP1, PIG55	**CTHT_0022620**	G0S4F4	YPL178W	Q08920	Cbc2
HGNC:7658	Q09161	**NCBP1**	CBP80, MGC2087, NCBP, Sto1	**CTHT_0061970**	G0SE05	YMR125W	P34160	Sto1
**hnRNP proteins**								
HGNC:5030	Q13151	**HNRNPA0**	HNRPA0	**CTHT_0055850**	G0SC42	YBR212W	P32831	Ngr1
HGNC:5031	P09651	**HNRNPA1**	HNRPA1, HNRPA1L3, MGC102835, ALS19	**CTHT_0062490**	G0SE55	YOL123W	Q99383	Hrp1
HGNC:24941	P51991	**HNRNPA3**	2610510D13Rik, D10S102, FBRNP	**CTHT_0062490**	G0SE55	YOL123W	Q99383	Hrp1
HGNC:5033	P22626	**HNRNPA2B1**	OPMD2, RNPA2, SNRPB1	**CTHT_0060190**	G0SEZ0	YGR159C	P27476	Nsr1
HGNC:5035	P07910	**HNRNPC**	HNRNP, HNRPC, MGC104306	**CTHT_0016200**	G0S271	YPL190C	P38996	Nab3
HGNC:5041	P31943	**HNRNPH1**	DKFZp686A15170, HNRPH, HNRPH1	**-**	-	-	-	-
HGNC:5045	P14866	**HNRNPL**	FLJ35509, HNRPL, P/OKcl.14, hnRNP-L	**-**	-	-	-	-
HGNC:8647	Q15365	**PCBP1**	hnRNP E1, HNRPE1, HNRPX	**CTHT_0027390**	G0S745	YBR233W	P38151	Pbp2
HGNC:9910	P38159	**RBMX**	HNRNPG, MRXSG, RBMXRT	**CTHT_0009820**	G0S0F3	-	-	-
**SR protein**								
HGNC:10781	P62995	**TRA2B**	DKFZp686F18120, Htra2-beta, SFRS10	**CTHT_0062190**	G0SE27	-	-	-
HGNC:10780	Q07955	**SRSF1**	ASF, FLJ53078, SF2, SFRS1	**-**	-	-	-	-
HGNC:16713	O75494	**SRSF10**	DKFZp686H0644, FLJ30749, FUSIP1	**-**	-	-	-	-
HGNC:10782	Q05519	**SRSF11**	DKFZp686M13204, FLJ18226, NET2, p54	**-**	-	-	-	-
HGNC:21220	Q8WXF0	**SRSF12**	FLJ14459, FLJ33484, FLJ41221	**-**	-	-	-	-
HGNC:10783	Q01130	**SRSF2**	PR264, SFRS2, SRp30b	**CTHT_0045250**	G0S9B7	-	-	-
HGNC:10785	P84103	**SRSF3**	SFRS3, SRp20	**CTHT_0045250**	G0S9B7	-	-	-
HGNC:10786	Q08170	**SRSF4**	SFRS4, SRP001LB, SRP75	**CTHT_0043780**	G0S8X5	YDR432W	Q01560	Npl3
HGNC:10787	Q13243	**SRSF5**	HRS, SFRS5, SRP40	**CTHT_0043780**	G0S8X5	YDR432W	Q01560	Npl3
HGNC:10788	Q13247	**SRSF6**	B52, FLJ08061, SFRS6, SRP55	**CTHT_0043780**	G0S8X5	YDR432W	Q01560	Npl3
HGNC:10789	Q16629	**SRSF7**	9G8, AAG3, SFRS7	**CTHT_0045250**	G0S9B7	-	-	-
HGNC:16988	Q9BRL6	**SRSF8**	DSM-1, SFRS2B, SRP46	**CTHT_0071140**	G0SFK3	YNL016W	P32588	Pub1
HGNC:10791	Q13242	**SRSF9**	SFRS9, SRp30c	**CTHT_0043780**	G0S8X5	YDR432W	Q01560	Npl3
HGNC:25482	Q9NWB6	**ARGLU1**	DKFZp686O08106, FLJ10154	**-**	-	-	-	-
