# Supplementary material for: Structural insights into spliceosome fidelity: DHX35–GPATCH1- mediated rejection of aberrant splicing substrates
Source: Cell Res. 2025 Feb 28;35(4):296–308. doi: 10.1038/s41422-025-01084-w (PMC11958768; doi:10.1038/s41422-025-01084-w)
Supplement: Supplementary file 17 — Supplementary information, Tables S3 [file 41422_2025_1084_MOESM17_ESM.pdf]

Table S3. Cryo-EM data collection, refinement and validation statistics.

	*ct*ILS	*ct*B*^{Q1}	*ct*B*^{Q2}	*ct*B*^{Q2} (focus DHX15)
**Data collection and processing**				
Magnification	130,000	130,000	130,000	130,000
Voltage (kV)	300	300	300	300
Electron exposure (e–/Å^2)	~50	~50	~50	~50
Defocus range (μm)	-1 to -2.5	-1 to -2.5	-1 to -2.5	-1 to -2.5
Pixel size (Å)	0.932	0.932	0.932	0.932
Symmetry imposed	*C1*	*C1*	*C1*	*C1*
Initial particle images (no.)	3,766,153	3,766,153	3,766,153	3,766,153
Final particle images (no.)	77,668	66,478	16,442	9,744
Map resolution (Å)	2.8	2.9	3.5	3.5
FSC threshold	0.143	0.143	0.143	0.143
Map resolution range (Å)	2.2-14	2.4-14	2.9-18	2.9-18
**Refinement**				
Model resolution (Å)	3.0	3.0	3.5	
FSC threshold	0.5	0.5	0.5	
Map sharpening *B* factor (Å^2)	-59	-63	-71	
Model composition				
Non-hydrogen atoms	104,129	87,156	92,486	
Protein residues	12,977	11,761	12,829	
RNA	341	265	260	
Ligands	9	7	7	
B factors (Å^2)	20.92	28.66	23.89	
Protein	20.34	27.72	23.29	
RNA	29.21	40.88	31.24	
Ligand	23.92	34.37	29.99	
R.m.s. deviations				
Bond lengths (Å)	0.007	0.003	0.006	
Bond angles (°)	0.856	0.702	0.788	
Validation				
MolProbity score	1.55	1.42	1.50	
Clashscore	5.71	4.86	4.91	
Poor rotamers (%)	0.02	0.02	0.03	
Ramachandran plot				
Favored (%)	96.38	97.03	96.40	
Allowed (%)	3.51	2.84	3.50	
Disallowed (%)	0.12	0.12	0.11	
**EMDB**	62841	62842	62843	62844
**PDB**	9L5R	9L5S	9L5T	
