# Supplementary material for: Structural insights into spliceosome fidelity: DHX35–GPATCH1- mediated rejection of aberrant splicing substrates
Source: Cell Res. 2025 Feb 28;35(4):296–308. doi: 10.1038/s41422-025-01084-w (PMC11958768; doi:10.1038/s41422-025-01084-w)
Supplement: Supplementary file 18 — Supplementary information, Tables S4 [file 41422_2025_1084_MOESM18_ESM.pdf]

Table S4. Protein composition of spliceosomal states presented in this study.

Human and yeast homolog proteins are given as in Table S1.

gene ID	Chain	B^{*Q1}	B^{*Q2}	ILS		Human	Yeast
**U2 snRNA**	**2**				U2 snRNA	**U2 snRNA**	U2 snRNA
**U5 snRNA**	**5**				U5 snRNA	**U5 snRNA**	U5 snRNA
**U6 snRNA**	**6**				U6 snRNA	**U6 snRNA**	U6 snRNA
**intron**	**7**				intron	**intron**	intron
**exon**	**8**				exon	**exon**	exon
**CTHT_0038260**	**a, j**				Sm	**E**	Sme1
**CTHT_0005760**	**b, l**				Sm	**F**	Smx3
**CTHT_0074540**	**g, k**				Sm	**G**	Smx2
**CTHT_0003330**	**d, o**				Sm	**D1**	Smd1
**CTHT_0022090**	**c, m**				Sm	**D2**	Smd2
**CTHT_0065260**	**f, u**				Sm	**D3**	Smd3
**CTHT_0072980**	**e, p**				Sm	**B/B'**	Smb1
**CTHT_0034680**	**h**				U2	**U2A'**	Lea1
**CTHT_0054050**	**i**				U2	**U2B"**	Msl1
**CTHT_0071250**	**A**				U5	**PRP8**	Prp8
**CTHT_0054900**	**C**				U5	**SNU114**	Snu114
**CTHT_0047230**	**E**				U5	**40K**	-
**CTHT_0052340**	**K**				PRP19/CDC5L	**SPF27**	SNT309
**CTHT_0038140**	**L**				PRP19/CDC5L	**CDC5L**	Cef1
**CTHT_0027480**	**P**				PRP19/CDC5L	**CWC15**	Cwc15
**CTHT_0008910**	**T**				PRP19/CDC5L	**PLRG1**	Prp46
**CTHT_0072540**	**q, r, s, t**				PRP19/CDC5L	**PRP19**	Prp19
**CTHT_0028670**	**0**				PRP19/CDC5L related	**RBM22**	Cwc2
**CTHT_0024880**	**B**				PRP19/CDC5L related	**SYF2**	Syf2
**CTHT_0010060**	**J**				PRP19/CDC5L related	**SYF3**	Syf3
**CTHT_0006330**	**M**				PRP19/CDC5L related	**RBM22**	Ecm2
**CTHT_0023130**	**N**				PRP19/CDC5L related	**BUD31**	Bud31
**CTHT_0061180**	**R**				PRP19/CDC5L related	**SKIP**	Prp45
**CTHT_0014950**	**S**				PRP19/CDC5L related	**PPIL1**	-
**CTHT_0033880**	**I**				IBC	**SYF1**	Syf1
**CTHT_0071530**	**Y**				IBC	**AQR**	-
**CTHT_0008120**	**CV**				IBC	**ISY1**	Isy1
**CTHT_0002650**	**Ci**				IBC	**PPIE**	-
**CTHT_0052020**	**F**				Bact proteins	**CCDC12**	-
**CTHT_0022970**	**V**				Bact proteins	**SRRM2**	Cwc21
**CTHT_0017830**	**W**				Bact proteins	**PRP17**	Prp17
**CTHT_0025210**	**Z**				Bact proteins	**CWC22**	Cwc22
**CTHT_0002590**	**z**				C* proteins	**DHX35**	-
**CTHT_0041140**	**1**				C* proteins	**GPATCH1**	-
**CTHT_0002620**	**D**				C* proteins	**SDE2**	-
**CTHT_0005780**	**Cc**				Disassembly	**DHX15**	Prp43
**CTHT_0074550**	**CY,Ck**				Disassembly	**GCFC2**	Ntr2
**CTHT_0020180**	**Cb**				Disassembly	**TFIP11**	Ntr1
**CTHT_0035950**	**U**				Disassembly	**CWF19L2**	-

RNA
core
catalytic
disassembly
