# Supplementary material for: Structural insights into spliceosome fidelity: DHX35–GPATCH1- mediated rejection of aberrant splicing substrates
Source: Cell Res. 2025 Feb 28;35(4):296–308. doi: 10.1038/s41422-025-01084-w (PMC11958768; doi:10.1038/s41422-025-01084-w)
Supplement: Supplementary file 19 — Supplementary figure legend [file 41422_2025_1084_MOESM19_ESM.pdf]

Figure S1: Cryo-EM data processing of the DHX15 sample.

a, Tandem-affinity purification of Protein A-TEV-FLAG-*ct*DHX15 from *C. thermophilum* lysates. The final FLAG-eluate was analyzed by SDS-PAGE and Coomassie-staining. Spliceosomal proteins were identified by mass spectrometry and labeled on the left according to their predicted molecular weights. The molecular weights (in kDa) of a protein standard are indicated on the right. **b**, Sorting scheme summarizing the data processing workflow for the DHX15 dataset. The masks, software and the key parameters used during various steps of data processing are indicated, alongside the schematic illustrations. Four classes were selected for final refinement and defined as B^{*Q1}, B^{*Q2}, B^{*Q2} (focus DHX15) and ILS complexes. The state B^{*Q2} (focus DHX15) map, and the map generated after focused classification of TFIP11 in state B^{*Q1}, were used to verify the rigid-body fit of DHX15 and TFIP11, respectively.

Figure S2: Cryo-EM structural analysis of DHX15 sample.

a, The local-resolution distributions and angle distributions of the four maps, as estimated by Relion and indicated by a blue-to-red color scale. **d**, Fourier shell correlation (FSC) curves of half-maps for ILS, B^{*Q1}, B^{*Q2} and B^{*Q2} (focus DHX15) complexes. The FSC cutoff criterion of 0.143 was used for corresponding resolution estimation. **e**, FSC curves comparing the model to the map for ILS, B^{*Q1} and B^{*Q2} complexes.

Figure S3: Structural details of the *ct*ILS state.

a, Two different views of the cryo-EM density maps of the ILS complex of *C. thermophilum* observed after affinity purification of DHX15. CWF19L1 is boxed and marked with an asterisk to indicate that its position cannot be assigned unambiguously. **b**, Models of the U2 (green), U5 (pink) and U6 (blue) snRNA with the intron-lariat RNA. A simplified scheme of the RNA interactions is illustrated in the bottom row. **c**, The resolution of the U2 and U6 snRNA, illustrated with the density maps, allows the identification of the bases in the 3' end of the U6 snRNA. The U2/U6 helix II and the poly-U stretch are indicated.

Figure S4: Structural comparison of the *ct*ILS complex with *ce*ILS".

a, Structure comparison between the *ct*ILS (left) and *ce*ILS” complexes (right, *C. elegans* ILS complex, PDB: 8I0R). Highlighted components: GCFC2 (red), TFIP11 (light blue), DHX15 (orange), SYF2 (pink), SDE2 (light green), PAXBP1 (red), CWF19L (purple), and PPIE (blue). *ct*ILS lacks CWF19L1 and PPIE, which are present in the *ce*ILS” complex. **b,** Close-up views of key regions in *ct*ILS (left) and *ce*ILS” (right) with focus on CWF19L1 (purple). In the ctILS state, CWF19L1 is boxed and marked with an asterisk to indicate that its position cannot be assigned unambiguously. **c**, Close-up views of key regions in *ct*ILS (left), *ce*ILS” (midddle) and scILS (right) with focus on RBM22 in *ce*ILS and ECM2/CWC2 in *ct* and *sc* ILS complexes.

Figure S5: Structural comparison of the DHX15 binding region in the *ct*ILS and *ce*ILS” complex.

a, Comparison between binding interface of DHX15 in the *ct*ILS (left) and *ce*ILS” complexes (right, *C. elegans* ILS complex, PDB: 8I0R). Highlighted components: DHX15 (orange), SYF2 (pink), SDE2 (green), SYF1 (light green) and CWF19L (purple). The U6 snRNA is shown in blue. **b**, Superimposition of DHX15 (orange) with the crystal structure of nucleotide-free DHX37 (gray) bound to RNA (yellow, PDB: 6O16). U6 snRNA is shown in blue.

Figure S6: Structural details of DHX35, GPATCH1, TFIP11, GCFC2, DHX15 and U2 snRNA from the B^{*Q1} complex.

a, Local resolution maps of DHX35 (left) and GPATCH1 (middle) in the B^{*Q1} complex, and DHX15 (right) in the *ct*ILS state. The color bars indicate the local resolution ranges for each protein as estimated by Relion. **b**, Close-up views of the cryo-EM densities for DHX35 (left, aa. 655-666), GPATCH1 (middle left, aa. 155-166), and GCFC2 (right, aa. 345-367), highlighting that the resolution is sufficient to visualize all side chain details. Models are shown as sticks surrounded by transparent density maps. In the middle right, the overall view of the density map generated after focused classification of TFIP11 in state B^{*Q1} shows the fit of TFIP11 into the density. **c**, The U2 and U6 snRNA are shown with the density maps. Interactions with PRP8, GPATCH1 and DHX35 are indicated.

Figure S7: Structural and functional insights into the spliceosomal 5′ splice site.

a, Density map of the active site center RNA in *ct*B^{*Q2} with the nucleotide positions of the modelled RNAs indicated. Detailed interaction between the U5 snRNA and 5' exon is shown with density map as an insert. **b**, Comparison of *ct*B^{*Q2} pre-mRNA with a pre-mRNA molecule from the B^{act} complex (PDB: 5Z56) illustrating the bulging loop. **c**, Functional validation of DHX35 and GPATCH1. Western blot analysis of endogenous Flag-FKBP12^{F36V} tagged DHX35 and GPATCH1 protein levels over time (0–4 hours) after dTAG-13 treatment demonstrates effective protein degradation (top left panel) in mouse ESCs. The DHX35 and GPATCH1 proteins were detected using Anti-Flag antibody. Anti-tubulin antibody was used to assess equal loading. **d-e**, Sequence logos depict the 5′SS motifs derived from RNA sequencing of mouse ESCs from wt (**d**) and DHX35 and GPATCH1 depleted cells (**e**). Comparisons reveal alterations in 5'ss sequence preferences, particularly from positions +3 to +6, highlighting the regulatory roles of DHX35 and GPATCH1 in splicing fidelity.

Figure S8: Suboptimal pre-mRNA in *ct*B^{*Q2} adopts an altered conformation in the active site center.

a, Close-up views of the splicing active center in *ct*B^{*Q2}, compared with two published states, *hs*B^{act} (PDB: 5Z56) and *hs*C (PDB: 6ZYM), showing different conformations of U2 and U6 snRNA. The U2 and BS helix undergoes a large conformational change to position the branch site adenosine (BS-A) in the active center in the B* complex (left to middle panel). The BS helix is released in the *ct*B^{*Q2} (right panel). **b**, Detailed view of the pre-mRNA in the splicing active center in *hs*B^{act} (PDB:5Z56) (left), *hs*C (PDB:6ZYM) (middle) and *ct*B^{*Q2} (right) complexes. The conformation of the pre-mRNA in *ct*B^{*Q2} spatially clashes with the BS-adenosine, placing *ct*B^{*Q2} in an inactive conformation. **c**, Schematic illustration of the RNA conformations within the active center in the predicted *ct*B^{act}, *ct*B^{*Q} and *ct*ILS active site centres. Due to missing information on the pre-mRNA, its sequence is represented as poly "N." The predicted RNA conformation in *ct*B^{act} is modeled based on the *hs*B^{act} structure (PDB: 5Z56).

Figure S9: Purification of protA-TEV-FLAG-tagged spliceosomal components TFIP11, GCFC2, DHX35, and WDR83.

a, SDS-PAGE analysis of the co-purified proteins using protA-TEV-FLAG-tagged TFIP11, GCFC2, DHX35, and WDR83 proteins. FLAG-tagged TFIP11, GCFC2,

DHX35, and WDR83 were expressed and purified from *C. thermophilum* using a similar approach as DHX15. The bait proteins are labeled. The purified samples were further analyzed by Mass spectrometry to identify co-purified proteins.

Figure S10: Structural comparison of the *ct*B*^{Q2}, *ct*ILS and *sc*ILS complexes.
a, Differences between *ct*B*^{Q2} (left), *ct*ILS (middle) and *sc*ILS (right) are highlighted: PRP8-RH domain (hot pink), GPATCH1 (green), DHX35 (tomato red), and CWF19 (green). **b**, Comparison of the position of the TFIP11-GCFC2 dimer in the *ct*B*^{Q2} (left), *ct*ILS (middle) and *sc*ILS (right) complexes. SRRM2 and CWC22 are present in *ct*B*^{Q2} (left) but missing in *ct*ILS (right). Notably, TFIP11 binds to a similar region in *ct*B*^{Q2} as it does in baker's yeast *sc*ILS state. **c**, Overlay of TFIP11 from *ct*B*^{Q2} with the position of DHX16 (left, PDB: 8CH6), DHX38 (middle, PDB: 5WSG) and DHX8 (right, PDB: 8C6J) during B^{act} and C* complexes. **d**, Close-up view of the steric clash between GCFC2 (left, aa 344-367) and the binding sites for DHX16 (aa 324-337, PDB: 8CH6), DHX38 (aa 371-384, PDB: 6ZYM) and DHX8 (aa 396-407, PDB: 8C6J).

Figure S11: Sequence alignment of GPATCH1.
a, Multiple sequence alignment of GPATCH1. Protein sequences from *C. Thermophilum* (*ct*), *N. Crassa* (*nc*), human (*hs*), *M. Musculus* (*mm*), *D. Melanogaster* (*dm*), *C. Elegans* (*ce*) were aligned using ClustalOmega. Interaction sites with PRP8 and DHX35 presented in this study are highlighted with bars. GPATCH1 W66 is indicated with red arrows. **b**, Multiple sequence alignment of G-patch domains from GPATCH1 variants compared to other G-patch proteins involved in splicing. Protein sequences from *C. Thermophilum* (*ct*), human (*hs*), *M. Musculus* (*mm*), *D. Melanogaster* (*dm*), *C. Elegans* (*ce*) and *S. pombe* (*sp*) were aligned using ClustalOmega. Brace-helix, insertion and brace-loop are indicated.

Figure S12: Domain architecture of key splicing components from *C. thermophilum*.

Figure S13: Molecular details of GPATCH1 binding to DHX35 and PRP8
a, The N-terminus of GPATCH1 binds to the C-terminal domain of DHX35 (DHX35-CTD). **b**, The region where GPATCH1 binds to DHX35 in *ct*B*^{Q2} spatially clashes with

the binding site of YJU2 in the *sc*B* complex (PDB: 6J6Q). **c**, Superimposition of the DHX35 (tomato) and GPATCH1 (forest green) complex with the crystal structure of DHX15 (gray) and SUGP1 (blue) (PDB: 8EJM). **d**, The middle region of GPATCH1 (aa 343-423) wraps around DHX35. **e**, A small stretch following the G-patch domain (aa 220-298) binds to the RT and linker domain of PRP8. **f**, the G-patch insertion forms interactions with the PRP8-RH domain. **g**, In the B^{*Q} complex, the PRP8-RH domain adopts a position close to the PRP8-linker. **h-k**, interactions between GPATCH1 and PRP8-EN. **l**, Rearrangements of the PRP8 domains during B^{act} to ILS transitions. **m**, The PRP8 a-finger is rearranged from B^{act} to C to accommodate the relocation of the U2/BS helix (left to middle panel). In B^{*Q2}, the α-finger adopts a conformation that more closely resembles its position in the B^{act} complex. **n**, Superimposition of GPATCH1 (*ct*B^{*Q2}) and the catalytic center of the C* complex (PDB: 8C6J) depicting clashes between the pre-mRNA and GPATCH1.

Figure S14: Structural details of the *ct*B^{*Q2} state.

a, Superimposition of DHX35 (tomato red) with the crystal structure of nucleotide-free DHX37 (gray) bound to RNA (yellow, PDB: 6O16). U2 snRNA from *ct*B^{*Q2} is indicated in green. **b**, Structural comparison of the splicing active center and U2/BS duplex with surrounding factors in *ct*B^{*Q2} (left), *sc*B* (middle, PDB: 6J6Q), and *hs*C (right, PDB: 8I0W). In the *ct*B^{*Q2} complex, the U2 snRNA is engaged with DHX35, and the BS has already been released. In the further progressed B* and C complexes, YJU2 and CWC25 stabilize the U2/BS helix during the first step of splicing. **c**, cryo-EM model of the docking site of DHX15 in the *ct*B^{*Q2} (left), *ct*ILS (middle) and *ce*ILS” (PDB:8RO1, right) complexes. Highlighted components: DHX15 (orange), SYF2 (pink), SDE2 (green), SYF1 (light green) and CWF19L (purple). The U6 snRNA is shown in blue. **d**, Comparison of U2/U6 helix II in relation to DHX15 in *ct*B^{*Q2}, *ct*ILS and *ce*ILS” (PDB: 8RO1). SYF2 and SDE2 form similar interactions, wrapping around the U2/U6 helix II.
